# Biomedical effects of protein arginine methyltransferase inhibitors

**DOI:** 10.1016/j.jbc.2025.108201

**Published:** 2025-01-16

**Authors:** Mengtong Cao, Terry Nguyen, Jiabao Song, Y. George Zheng

**Affiliations:** Department of Pharmaceutical and Biomedical Sciences, University of Georgia, Athens, Georgia, United States

**Keywords:** PTM, PRMT, protein arginine methylation, epigenetics, MTA, MTAP, inhibitor, drug discovery, pharmacology

## Abstract

Protein arginine methyltransferases (PRMTs) are enzymes that catalyze the methylation of arginine residues in eukaryotic proteins, playing critical roles in modulating diverse cellular processes. The importance of PRMTs in the incidence and progression of a wide range of diseases, particularly cancers, such as breast, liver, lung, colorectal cancer, lymphoma, leukemia, and acute myeloid leukemia is increasingly recognized. This underscores the critical need for the development of effective PRMT inhibitors as therapeutic intervention. The field of PRMT inhibitors is in the rapidly growing phase and it is necessary to conduct a summative review of how the so-far developed inhibitors impact PRMT functions and cellular physiology. Our review aims to summarize molecular action mechanisms of these PRMT inhibitors and particularly elaborate their triggered biomedical effects. We describe the cellular phenotype consequences of select PRMT inhibitors across various disease models, thereby providing an understanding of the pharmacological mechanisms underpinning PRMT inhibition. The promising effects of PRMT5 inhibitors in targeted therapy of methylthioadenosine phosphorylase-deleted cancers are particularly highlighted. At last, we provide a perspective on the challenges and further opportunities of developing and applying novel PRMT inhibitors for clinical advancement.

Covalent posttranslational modifications of proteins are involved in the regulation of multifaceted biological processes in cells such as transcription, translation, protein-protein interaction, signal transduction, and RNA metabolism (1, 2). On the chromatin, histone modification marks are abundant and often act synergistically with each other, with one modification influencing the addition or removal or reading of another on the same or different histone proteins, a concept well-known as the “histone code” (3, 4). Such interplays can lead to complex effects of epigenetic modulators, altering structural dynamics of the nucleosomes and gene expression patterns (5). Among the diverse posttranslational modifications, the addition of a methyl group to proteins represents a prominent chemical modification that occurs on the side chain of a variety of amino acid residues including arginine, lysine, histidine, glutamate, glutamine, asparagine, and cysteine (6, 7, 8, 9). Protein arginine methyltransferases (PRMTs) are the enzymes that specifically catalyze the methylation of arginine residues in which the methyl group from the methyl donor *S*-adenosyl-L-methionine (SAM, AdoMet) is transferred to the terminal guanidino nitrogen of an arginine residue’s side chain (10). Currently, nine PRMTs have been identified in mammalian cells and they are classified into type I, type II, and type III according to their catalytic activity. Type I PRMTs consists of PRMT1, −2, −3, −4 (CARM1), −6, and −8. PRMT5 and PRMT9 are grouped into type II enzymes and PRMT7 is the only type III PRMT enzyme. All the three types of enzymes are able to transfer one methyl group from SAM to arginine residue to produce *N*^*G*^-monomethyl arginine (MMA, Rme1) (Fig. 1*A*). Differentially, type I PRMTs add one more methyl group to MMA and generate asymmetric *N*^*G*^, *N*^*G*^-dimethylarginine (ADMA, Rme2a), while type II PRMTs add a second methyl group symmetrically to MMA and produce *N*^*G*^, *N’*^*G*^-dimethylarginine (SDMA, Rme2s) (11, 12).

**Figure 1:**
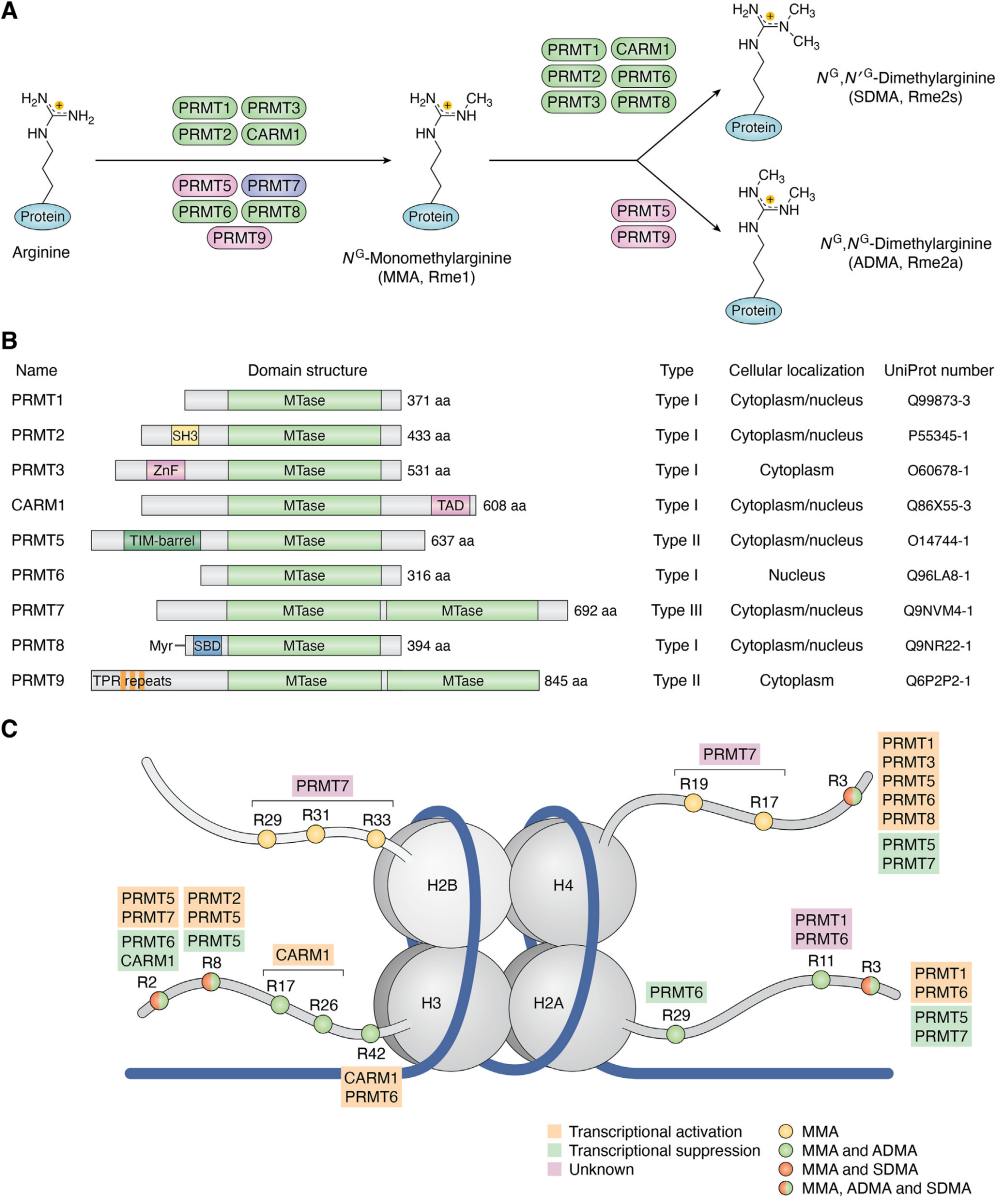
**PRMTs and protein arginine methylation.***A*, the structures and types of PRMT family members, key structures are indicated including MTase domain. *B*, MMA, ADMA, and SDMA are three types of arginine methylation catalyzed by different types of PRMTs. The protein sequences can be found in UniProt: PRMT1, Q99873-3; PRMT2, P55345-1; PRMT3, O60678-1; PRMT4/CARM1, Q86X55-3; PRMT5, O14744-1; PRMT6, Q96LA8-1; PRMT7, Q9NVM4-1; PRMT8, Q9NR22-1; PRMT9, Q6P2P2-1. *C*, histone substrates of PRMTs and their regulation of gene expression. The methyltransferases are responsible for adding methyl groups to specific arginine (R) residues on histone tails. The methylation has a significant impact on transcriptional regulation. The consequences of these methylations and their influence in controlling gene expression are indicated. PRMT, protein arginine methyltransferase; MMA, *N*^*G*^-monomethyl arginine; ADMA, asymmetric dimethylarginine; SDMA, symmetric dimethylarginine; MTase, methyltransferase.

All members of the PRMT family share a conserved methyltransferase (MTase) domain, which is crucial for SAM binding and substrate recognition (Fig. 1*B*). PRMTs have four conserved motifs contained in the MTase domain: motif I (VLD/EVGXGXG), post I (V/IXG/AXD/E), motif II (F/I/VDI/L/K), and motif III (LR/KXXG) (13). Despite this commonality, each PRMT family member has significant variations in its primary sequences and some even contain unique intact structural domains. For instance, PRMT2 possesses an Src homology 3 (SH3) domain at its amino terminus, while PRMT3 is characterized by a zinc finger (ZnF) domain (14, 15). CARM1 includes a transcriptional activation domain (TAD) on its carboxyl terminus, and PRMT5 features a TIM-barrel domain at its N terminus (16, 17). In contrast to other PRMTs that contain a single MTase domain, PRMT7 and PRMT9 each have two tandem MTase domains. PRMT8 is distinguished by the presence of a myristoylation motif at its N terminus, while PRMT9 contains three tetratricopeptide repeat (TPR) motifs at its N-terminal region (18, 19, 20).

JMJD6 and JMJD1B are critical regulators of arginine methylation, functioning as arginine demethylases (21). JMJD6 is an Fe (II)- and 2-oxoglutarate-dependent dioxygenase. The mechanism begins with JMJD6 catalyzing the hydroxylation of the methyl group, a reaction that consumes oxoglutarate (2OG). This initial step is followed by a deformylation reaction, which generates formaldehyde (CH₂O) as a byproduct (22). JMJD1B has been reported to demethylate H4R3me2s and its intermediate H4R3me1, promoting gene expression crucial for the development of hematopoietic stem and progenitor cells (23). PAD4 is a peptidylarginine deiminase; it could convert monomethylated arginine residues to citrulline through a Ca^2^⁺-dependent process. The activity of PAD4 modulates histone arginine methylation, impacting gene expression by demethylating histones both *in vitro* and *in vivo*. This enzyme specifically targets histones H3 and H4 at multiple sites, including methylation sites established by coactivators such as CARM1 (H3R17) and PRMT1 (H4R3), thereby playing a crucial role in chromatin dynamics and transcriptional regulation (24). Furthermore, methylation readers, especially Tudor domain proteins, *e.g.*, survival of motor neuron (SMN), SPF30, TDRD3, and TDRKH, recognize methylated arginines in substrates and play regulatory roles in RNA splicing, gene expression, and small RNA silencing (25, 26, 27). For instance, SMN recognizes arginine methylation marks through its Tudor domain, which allows it to engage with Sm proteins that are essential for spliceosome assembly, impacting both splicing and transcriptional termination. Similarly, SPF30 binds to SDMA motifs, supporting RNA decay and ribosome biogenesis. Additionally, TDRD3 is known for its role in resolving R-loops during transcription by interacting with ADMA marks on histones, while TDRKH has been shown to interact with PIWI proteins, thereby participating in RNA silencing pathways (28). Recently it was found that non-Tudor domain protein SART3 recognizes SDMA-marked GAR motifs and thereby affects RNA splicing (29).

A large number of protein substrates of PRMTs in the cell are nucleic acid-binding proteins (1). Thus, not surprisingly, nuclear histones are important substrates of PRMTs, and different PRMTs exhibit distinct site specificity, such as H2AR11 (PRMT1 and PRMT6), H2BR29 (PRMT7), H3R2 (PRMT5 and PRMT6), H3R8 (PRMT5), H3R17 (CARM1), H3R26 (CARM1), and H4R3 (PRMT1, -5, and PRMT6) (30, 31, 32, 33, 34) (Fig. 1*C* and Table S1). A previous study reported that H4R3me1 is present on only about 0.2% of histone H4 molecules, reflecting its low stoichiometric abundance (35). In contrast, H4K20me2 is found on approximately 70 to 80% of H4 molecules. This difference suggests distinct mechanistic roles: the limited abundance of H4R3me1 implies specialized regulatory functions, whereas the widespread presence of H4K20me2 is indicative of its broader role in chromatin organization and gene regulation. Arginine methylation generally promotes gene expression, although certain modifications can also act as repressors. Most arginine methylations are associated with the activation of gene expression while a few other methylations suppress gene expression (36) Interestingly, PRMT5-mediated symmetric di-methylation on histone H4R3 and H3R8 is generally regarded as a repressive epigenetic mark, contributing to gene silencing. However, under specific conditions, PRMT5 can also promote transcriptional activation of target genes, demonstrating its context-dependent role in gene regulation (37, 38). Prominently, PRMTs have a broad spectrum of nonhistone substrates, contributing significantly to various nuclear and nonnuclear biological processes including transcription, RNA splicing, DNA repair, and signal transduction (39). PRMT1, -3, -5, and -8 display a preference for methylating glycine(G)- and arginine(R) rich (GAR) motifs (15, 19, 40, 41). PRMT2, though having little activity *in vitro*, has been identified to methylate proteins with proline(P)-rich motifs as well as serine (S)- or arginine (R)- rich motifs (42). CARM1 exhibits a distinct preference for methylating proline(P)-, glycine(G)- and methionine(M)-rich motifs (43). PRMT7 has been found to recognize RXR motifs, where X represents a variable residue (44). Interestingly, PRMT9 demonstrates minimal to no activity concerning core histones or proteins containing GAR motifs (18).

The subcellular localization of PRMTs is critical in governing their functional regulations. Certain PRMT enzymes have been shown to shuttle between the nuclear and cytoplasmic compartments, participating in signal transduction pathways and RNA metabolism (45). Specifically, PRMT1, −2, -3, -4, and 7 are found in both the cytoplasm and nucleus, while PRMT6 in human embryonic kidney (HEK293) cell lines was found exclusively in the nucleus and PRMT5 was found only in cytoplasm of HEK293 cells. Additionally, PRMT8 was found at the plasma membrane of HEK293 cells (46). Nevertheless, subcellular localization of PRMTs could be cell and tissue type dependent, though its regulatory factors are very little explored.

The research into protein arginine methylation has increasingly underpinned the pivotal roles of PRMTs in oncology, highlighting the urgency to develop effective PRMT inhibitors for cancer therapy (47, 48). The upregulation of PRMT1, -2, -4, -5, and -7 is related to the progression of breast cancer cells (49, 50, 51, 52, 53). Similarly, in hepatocellular carcinoma (HCC), the expression of PRMT1, -5, and -9 has been correlated with disease advancement, suggesting these as potential therapeutic targets (54, 55, 56). In lung cancer, PRMT1, -5, -6, and -7 have been identified as key players (57, 58, 59, 60, 61). Additionally, PRMT1, -4, and -5 have been implicated in the progression of colorectal cancer (CRC) (62, 63, 64). Also, PRMT5 is particularly noteworthy for its association with lymphoma, leukemia, and acute myeloid leukemia (AML) (65, 66, 67). These findings collectively signify the critical importance of PRMTs in the pathophysiology of a wide spectrum of cancers, paving the way for the development of PRMT-targeted therapeutic strategies.

Since the first report from Cheng et al., on small molecule inhibitors for arginine methylation in 2004 (68), the field of PRMT drug discovery has witnessed remarkable progress. This advancement is exemplified by the development of a number of small molecule inhibitors, with micromolar or nanomolar efficacies, targeting various PRMT (69, 70, 71, 72). Notable examples include diamidines for PRMT1, SGC707 for PRMT3, and EPZ015666 for PRMT5 (73, 74). A list of representative small molecule PRMT inhibitors is presented in Table 1. The field is evolving quickly, manifested by a significant number of emerging patents and notable biological discoveries. Among these discoveries is the identification of key PRMT enzymes, such as PRMT5, which are recognized as viable drug targets. Therefore, quite a few comprehensive reviews about PRMT inhibitor development have been available in the literature (71, 75, 76). This review is purposed to provide an up-to-date on the field with the latest advancements. In particular, we review and assay the reported biomedical effects of select representative PRMT inhibitors in different disease models, which offer mechanistic insights and understanding of PRMT inhibitor pharmacology and toxicology. The significance of PRMT inhibitors in biomedical research and therapy is profound, offering a promising frontier for the treatment of various diseases. The inhibitors included in this essay stand out for their multifaceted biological effects, which span anti-inflammatory, antifibrotic, and anticancer activities. Their ability to modulate key cellular processes and signaling pathways highlights the potential for targeted therapies that could provide more effective and less toxic alternatives to current treatment modalities.

**Table 1:** Structures, targets and IC_50_ of PRMT inhibitors

Compound	Structure	Target protein	IC_50_	Year of report
AMI-1		PRMT1	8.8 μM	2004 (68)
		CARM1	74 μM	2012 (77)
MS023		PRMT1	30 nM	2016 (88)
		PRMT3	119 nM	
		CARM1	83 nM	
		PRMT6	4 nM	
		PRMT8	5 nM	
GSK3368715		PRMT1	3.1 nM	2019 (96)
		PRMT3	162 nM	
		CAMR1	38 nM	
		PRMT6	4.7 nM	
		PRMT8	3.9 nM	
TC-E 5003		PRMT1	1.5 μM	2011 (100)
DB75		PRMT1 PRMT5	9.4 μM 166 μM	2014 (73)
K313		PRMT1	2.6 μM	2021 (104)
		PRMT3	3.4 μM	
		CAMR1	27.6 μM	
		PRMT5	6.8 μM	
		PRMT6	2.6 μM	
		PRMT7	23.6 μM	
		PRMT8	8.2 μM	
EPZ015666		PRMT5	22 nM	2015 (120)
GSK591		PRMT5	4 nM	2016 (131)
LLY-283		PRMT5	22 nM	2018 (138)
SGC707		PRMT3	31 nM	2015 (74)
EZM2302		CARM1	6 nM	2017 (148)
TP064		CARM1	<10 nM	2018 (152)
		PRMT6	1.3 μM	
		PRMT8	8.1 μM	
MS049		CARM1 PRMT6	34 nM 43 nM	2016 (154)
MS117		PRMT6	18 nM	2020 (157)
SGC6870		PRMT6	77 nM	2021 (158)
SGC3027		PRMT7	1.3 μM	2020 (160)

## AMI-1

Arginine methyltransferase inhibitor 1 (AMI-1) is the first discovered PRMT inhibitor (68). It is a pan PRMT inhibitor that decreases the enzymatic activity of PRMT1, PRMT3, PRMT4, PRMT5, and PRMT6. The half-maximum inhibitory concentration (IC_50_) value of AMI-1 against recombinant PRMT1 enzyme varies from 8.8 to 137 μM, depending on chosen experimental conditions. AMI-1 also inhibits CARM1 activity, with an IC_50_ of 74 μM (77). Some key functional groups found on AMI-1 include two naphthyl rings located on either side of the central urea. It is thought that the urea may mimic the guanidine found on the arginine side chain while the naphthyl rings are approximately the same length as the alkyl chain leading to the guanidino group. Furthermore, the sulfonate substituents found on the naphthyl ring was theorized to mimic the carbonyl from arginine, though with all of this, the binding mechanism of AMI-1 has not been confirmed. The authors performed a UV-crosslinking experiment which showed that AMI-1 did not compete with SAM binding suggesting that the inhibitor instead binds with the substrate pocket of PRMT1. However, docking studies refute the claim that the inhibitor targets the substrate binding site as more favorable poses tended to have AMI-1 bound to the SAM pocket. As this was shown experimentally to not be the case, another method must be used to validate the inhibitor’s mode of binding. However, due to the highly charged sulfonates, it is likely that AMI-1 will have poor bioavailability leading to the desire to develop analogs to this probe (68). The radioisotope-labeled biochemical assay showed that AMI-1 is a linear competitive inhibitor *versus* H4 or GAR peptide, while is noncompetitive *versus* AdoMet (78). Importantly, detailed biochemical and biophysical studies illustrated that AMI-1 and similar organic compounds bearing naphthalene and sulfonyl groups can directly target the highly positively charged histone or GAR peptide substrates, and the binding subsequently blocks the recognition of the substrates by the PRMT enzyme (79). This mechanism provides another layer of reasonable explanation for its pan inhibitory activity against PRMTs (80).

**Figure 2:**
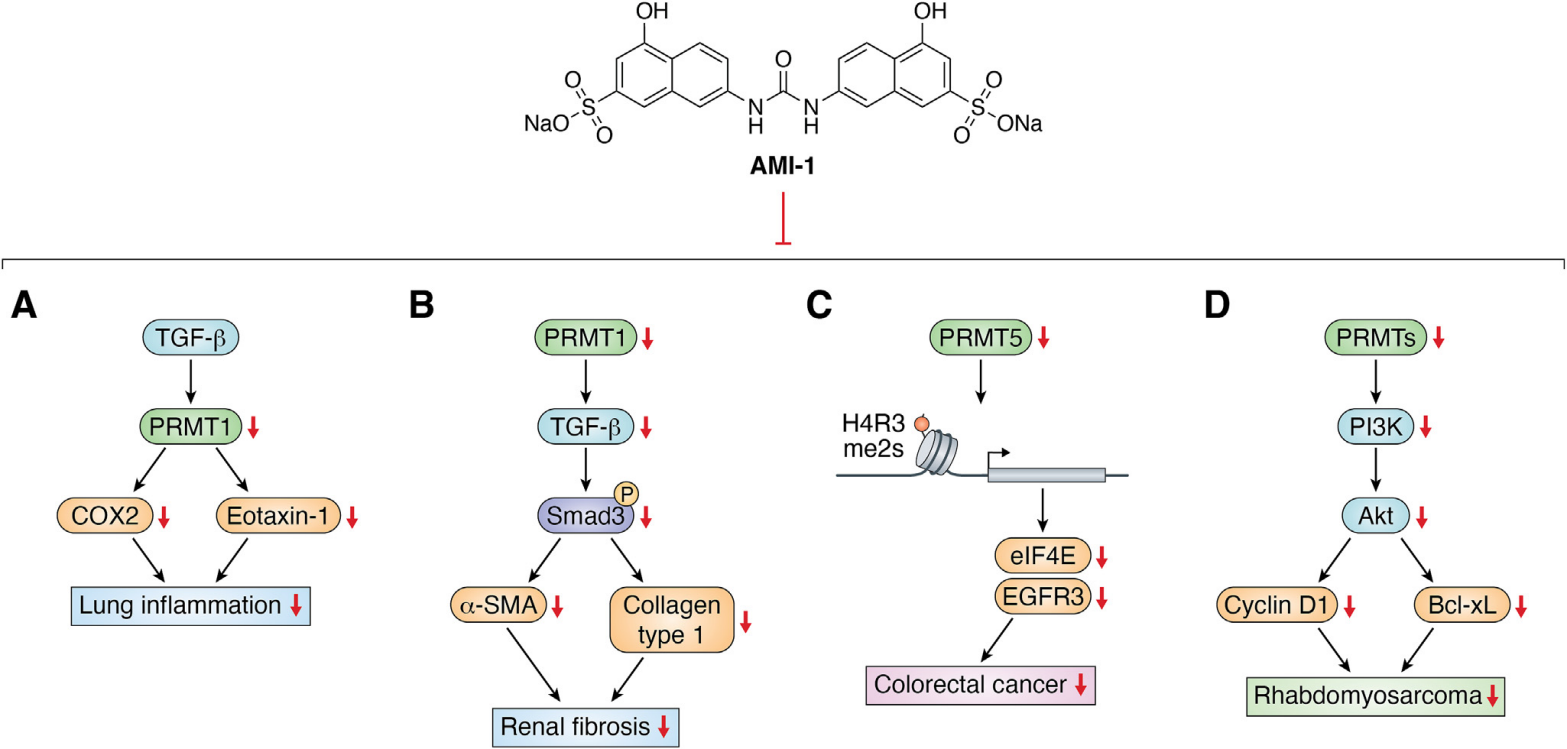
**The mechanistic implication of AMI-1 as an oncologic therapeutic including lung inflammation, reno fibrosis, colorectal cancer, and rhabdomyosarcoma.***A*, AMI-1 reduces inflammation by suppressing PRMT1, COX2, and eotaxin-1, thereby alleviating chronic Ag-induced pulmonary inflammation. *B*, AMI-1 has a kidney-protective effect by halting renal fibrosis progression in mice through the reduction of fibrosis markers like Smad3 phosphorylation and TGF-βRI expression in kidney fibroblasts. *C*, AMI-1 impedes colorectal cancer cell proliferation and migration by targeting PRMT5. The H4R3me2s level of eIF4E and EGFR is decreased and the expression level of the factors is decreased. *D*, AMI-1 reduces rhabdomyosarcoma (RMS) cell growth and promotes apoptosis through its suppression of the PI3K-Akt pathway. PRMT, protein arginine methyltransferase; AMI-1, arginine methyltransferase inhibitor 1; FGFR, fibroblast growth factor receptor; TGF-βRI, transforming growth factor-beta receptor I.

AMI-1 is reported to exhibit the antiinflammatory activity. The administration of AMI-1 in rat lungs and A549 cells inhibited PRMT1 expression, further downregulating the expression of COX2 and eotaxin-1 to ameliorate chronic Ag-induced pulmonary inflammation (Fig. 2*A*) (81, 82). Another therapeutic effect of AMI-1 is reno-protective effect, where its administration significantly curbed the progression of renal fibrosis in a murine model. By attenuating key fibrotic markers, such as Smad3 phosphorylation and transforming growth factor-beta receptor I (TGF-βRI) expression in renal interstitial fibroblasts, AMI-1 illustrates promising results in reducing renal fibrosis (Fig. 2*B*) (2).

AMI-1 also targets PRMT5, showing its potential in oncological therapeutics. Studies have revealed that AMI-1 instigates cell cycle arrest and triggers apoptosis across various cancer cell lines. AMI-1 inhibits CRC cell proliferation and migration. AMI-1-mediated inhibition of PRMT5 decreased H4R3me2s methylation at the promoters of EGFR3 and eIF4E, resulting in reduced expression of both genes (Fig. 2*C*) (83). Additionally, combining AMI-1 with cisplatin can effectively decrease cell viability, induce cell growth arrest and apoptosis in lung adenocarcinoma cells. This approach shows potential for improving cisplatin resistance in lung cancer. Moreover, the scope of AMI-1's impact extends beyond lung and CRCs, as its inhibitory action on PRMT5 plays a crucial role in combating other malignancies, including gastric, cervical, and hepatocellular (83, 84, 85, 86).

The PRMTs are recognized for their elevated expression in rhabdomyosarcoma (RMS) cell lines. The *in vitro* and *in vivo* assays have corroborated that AMI-1 significantly curtails RMS cell proliferation and propels apoptotic processes. This inhibitory effect is likely linked to the dampening of the PI3K-Akt signaling pathway, a crucial regulator of cell survival and growth. By inhibiting PRMTs and impeding this pathway, AMI-1 effectively undermines the cellular mechanisms that contribute to the pathogenesis and progression of RMS (Fig. 2*D*) (87).

## MS023

**Figure 3:**
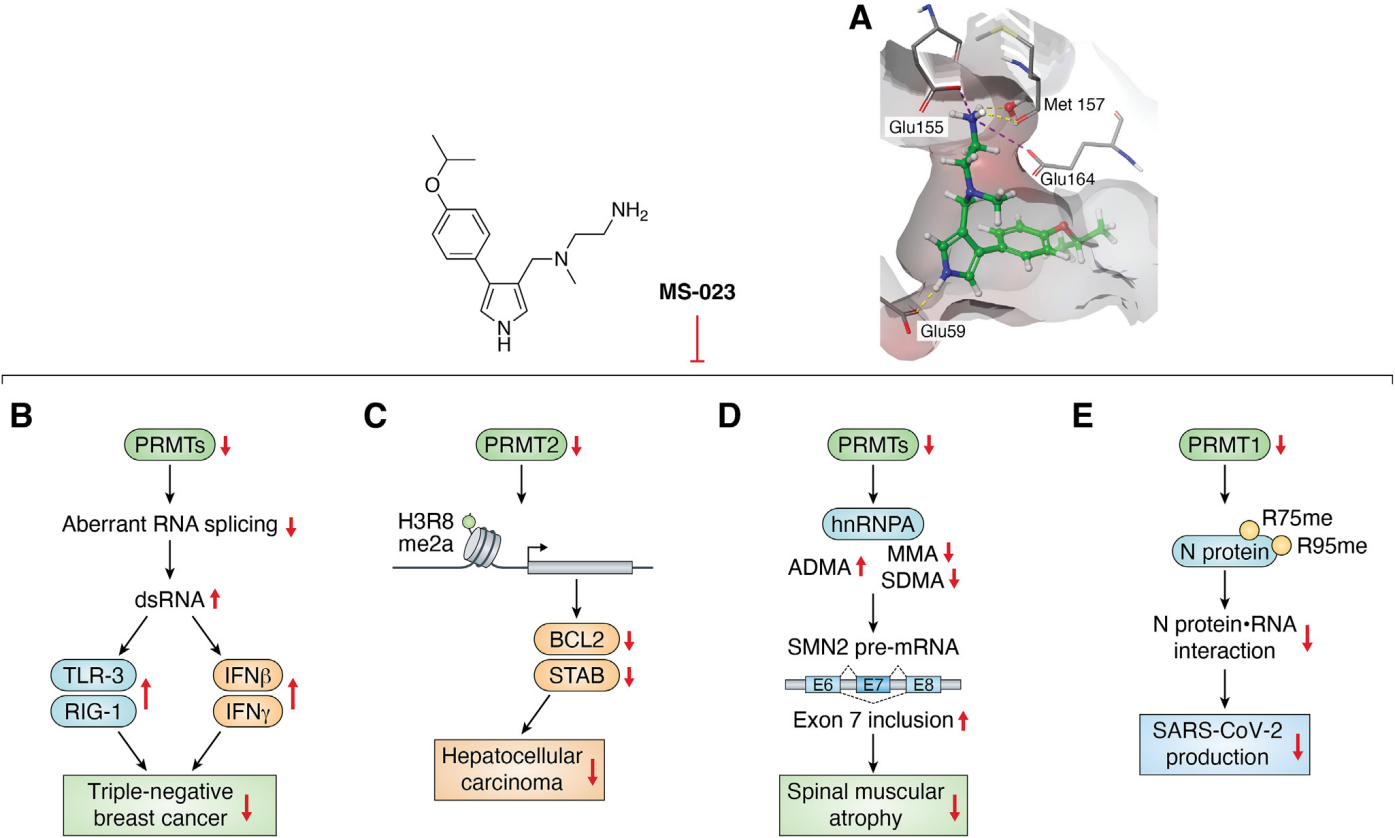
**Biomedical effect of MS023.***A*, MS023 in PRMT1 (PDB:5E8R). *Green* structure: MS023; *Yellow dashes*: hydrogen bonds. *Pink dashes*: ionic interactions. *B*, MS023 inhibits PRMTs from triggering a viral mimicry response in TNBC cells, causing intron retention and dsRNA accumulation that leads to increased expression of interferon-responsive and dsRNA-sensing genes. *C*, MS023 inhibits growth and induces apoptosis in hepatocellular carcinoma cells by targeting PRMT2, which regulates BCL2 expression through H3R8me2a methylation. *D*, MS023 enhances the therapeutic approach to spinal muscular atrophy (SMA) by specifically inhibiting hnRNPA1 binding, a pivotal action that promotes the inclusion of exon 7 in the SMN2 transcript. *E*, MS023 disrupts the interaction between the N protein and the 5′-UTR of SARS-CoV-2 RNA by inhibiting type I PRMTs that significantly reduces viral replication in VeroE6 cells. PRMT, protein arginine methyltransferase; TNBC, triple-negative breast cancer; SMN2, survival of motor neuron 2; hnRNPA1, heterogeneous nuclear ribonucleoprotein A1.

MS023 is a potent inhibitor of type I PRMTs, exhibiting a robust inhibitory activity for PRMT1, 3, 4, 6, and 8 with IC_50_ values of 30 ± 9 nM, 119 ± 14 nM, 83 ± 10 nM, 4 ± 0.5 nM, and 5 ± 0.1 nM, respectively (88). The crystal structure of MS023 bound to PRMT6 (Protein Data Bank (PDB) 5E8R) revealed the compound's ability to interact with the substrate binding site (88). The amines form hydrogen bonds with Glu155, Met157, and Glu164. Other important interactions include the hydrogen bond formed between the nitrogen in the pyrrole ring with Glu59, the pi-pi interaction of the phenyl ring with Tyr159 and the hydrogen bond of His163 by the isopropoxy group (Fig. 3*A*). Biochemical tests demonstrated that the inhibitor displays noncompetitive inhibition with regards to the cofactor SAM for type I PRMTs except in the case of PRMT3, for which MS023 appears to be noncompetitive with the peptide substrate but uncompetitive with SAM. It was theorized that this could be due to a difference in conformational change of the PRMTs with MS023 as compared to substrates. Through the structure-activity relationship (SAR) studies, it was found that the amines on the alkyl chain are important for binding as altering these atoms to either oxygen or carbonyl reduces potency (88). Along these lines, conversion of the pyrrole to a 1,2,3-triazole reduces potency quite significantly. From earlier compounds containing a meta position CF_3_ group, replacing this substituent with a para-isopropoxy group greatly improves the potency of the compound. Cellular studies showed that MS023 reduces global levels of ADMA while enhancing the levels of MMA and SDMA (88).

Type I PRMTs are key mediators of malignancy in tumor progression, which brings MS023 as a potential cancer drug candidate. As the predominant type I member, PRMT1 has been identified as a crucial facilitator of growth in triple-negative breast cancer (TNBC) (89). Significantly, MS023 is shown to invoke a viral mimicry response in TNBC cells (Fig. 3*B*). This intriguing mechanism involves intron retention and consequent accumulation of dsRNA, a molecular pattern typically associated with viral infections. The downstream effect of this is a pronounced upregulation of interferon (IFN)-responsive genes, such as IFNβ and IFNγ, alongside genes integral to the dsRNA-sensing pathway, including STING1 and TLR-3. Notably, the therapeutic potential of MS023 seems to be contingent upon a preexisting heightened expression of IFN response genes within TNBC cells (89). In addition to the inhibitory effects on TNBC cells, MS023 was found to inhibit cell growth and induce apoptosis in HCC cells (90). Notably, PRMT2 has been implicated in the regulation of expression within HCC cells, acting through the methylation of H3R8me2a at Bcl2 (encoded by BCL2 apoptosis regulator gene) promoter, increasing Bcl2 and Stab gene expression (Fig. 3*C*). The study has demonstrated MS023 inhibits PRMT2 activity and disrupts its oncogenic function (90). Moreover, MS023 targets PRMT1, effectively inhibits colon cancer cells by inducing differentiation changes, as demonstrated by proteomic and genomic analyses in the aggressive HT-29 cell line. MS023 treatment significantly delayed HT-29 xenograft growth in nude mice, showing potential for clinically effective anticancer therapy through cancer cell differentiation (91).

Converging evidence from multiple studies suggests that the efficacy of MS023 can be substantially enhanced when used in conjunction with other therapeutic agents. Notably, its combination with poly-ribose polymerase (PARP) inhibitors has shown promising results in specific nonsmall cell lung cancer cell lines (92). As one of the PARP inhibitors, BMN-673—a Food and Drug Administration-approved drug—exhibits a particularly potent synergistic effect when paired with MS023 in methylthioadenosine phosphorylase (MTAP)-negative A549 cells. This synergism is mechanistically grounded in the augmentation of DNA damage, as the combination treatment notably amplifies γ-H2AX foci accumulation—a sensitive marker of DNA damage and repair— thereby increasing cytotoxicity in A549 cells (92). Another combination treatment is the recent investigation by Zhang *et al.* (93) into the therapeutic potential of combining type I PRMT inhibition with anti-PD-1 immunotherapy in TNBC. They found that this combination reduced tumor growth by reshaping the tumor microenvironment, increasing CD8 T-cell infiltration, and enhancing T-cell repertoire diversity, suggesting improved immunotherapy outcomes in TNBC. Moreover, MS023 acts as a promising therapeutic candidate for spinal muscular atrophy (SMA), a primary genetic cause of infant mortality. Kordala *et al.* (94) reported the potential of MS023 as both a standalone and adjunctive treatment for SMA. Their findings revealed that MS023 significantly mitigates the SMA phenotype in preclinical models. Such a therapeutic effect is seemingly attributed to its inhibition of heterogeneous nuclear ribonucleoprotein A1 (hnRNPA1) binding, thereby facilitating the inclusion of exon 7 in the SMN2 transcript and leading to the production of the full-length SMN2 protein, which is essential for combating SMA. The efficacy of MS023 is also notably amplified when combined with the SMN2-targeting antisense oligonucleotide nusinersen, the combination of which has been demonstrated to exert a synergistic effect, markedly enhancing the correction of the SMA transcriptomic signature in SMA mouse models (Fig. 3*D*) (94).

Cai *et al.* (95) uncovered that the SARS-CoV-2 nucleocapsid (N) protein contains five RGG/RG motifs, which are preferential substrates for PRMTs. Their research further elucidated that PRMT1 specifically methylates the N protein at the R95 and R177 residues within these motifs. As an inhibitor of type I PRMTs, MS023 significantly alters the interactome of the N protein. Crucially, MS023 impedes the interaction between the N protein and the 5′-UTR of SARS-CoV-2 genomic RNA, a pivotal process for the virus's packaging and assembly into virions (Fig. 3*E*). Notably, they demonstrated that pretreatment of VeroE6 cells with MS023 leads to a substantial reduction in SARS-CoV-2 replication, marking the potential of PRMT inhibition in host cells for combating coronavirus infection (95).

## GSK3368715

GSK3368715 is a type I PRMT inhibitor structurally analogous to MS023 (Ki_app_ values ranging from 1.5 to 81 nM) (96). It binds to the substrate binding pocket of the enzymes and exhibits a noncompetitive inhibition with regards to SAM. GSK3368715 displays a strong interaction between the side chain of Glu162 of PRMT1 with its secondary amine on the alkyl chain. This amine also has hydrogen bonds with the backbone carbonyl of Met164. The tertiary amine of the compound hydrogen bonds with His311. There is a moderate interaction between the secondary amine of the diazole and Glu65. The ethoxy substituents interact with Tyr170 *via* a water molecule-mediated interaction.

GSK3368715 has demonstrated antitumor activity across a range of tumor types. The preclinical evidence indicates that GSK3368715 triggers a decrease in ADMA levels after 72 hours and strong antiproliferative activity in a range of solid and hematological malignancies (97). The critical mechanism is that GSK3368715 leads to alternation of RNA splicing which further causes aberrant exon usage. In colorectal cancer, GSK3368715 exhibits significant antitumor effects by targeting PRMT1. PRMT1-mediated arginine methylation of PGK1 at R206 enhances glycolysis and tumorigenesis through extracellular signal-regulated kinase (ERK)-mediated phosphorylation at S203. Inhibition of PRMT1 with GSK3368715 reduces glucose uptake, lactate production, and extracellular acidification rates, suppressing tumor growth and progression in CRC models (98). Furthermore, it exhibits antiproliferative effects particularly when combined with PRMT5 inhibitor GSK3203591 in pancreatic cancer and diffuse large B-cell lymphoma cell lines (96). These promising therapeutic potentials led to the progression of GSK3368715 into phase I clinical trials. Unfortunately, the higher-than-expected incidence of thromboembolic event caused the termination of the clinical study (99).

## TC-E 5003

N,N′-(Sulfonyldi-4,1-phenylene)bis(2-chloroacetamide), commonly referred to as TC-E 5003 (TC-E), was reported to be a specific inhibitor of PRMT1 (100). It was discovered through a screening based on the lead compound allantodapsone. In docking simulations, TC-E 5003 was found to interact through its aryl amine with Glu152 in the target pocket. With SAR studies, it was observed that diacylated compounds generally provided for an increase in potency except in the case of the propionamide analog. Among the various derivatives, TC-E 5003 proved to be the most potent compound exhibiting an IC_50_ of 1.5 ± 0.2 μM (100). As a chemical probe of PRMT1, TC-E 5003 was applied to test the roles of PRMT1 in modulating various physiological and pathological processes.

Recent research shed light on the remarkable antiinflammatory properties of TC-E 5003 (101). Mechanistically, TC-E 5003 inhibits PRMT1, reducing lipopolysaccharide (LPS)-mediated nitric oxide production and curtails the expression of inflammatory genes. This inhibition extends to the nuclear translocation of NF-κB subunits p65 and p50, as well as the AP-1 transcription factor c-Jun. Intriguingly, TC-E also directly influences c-Jun gene expression following LPS exposure. In the context of NF-κB signaling, TC-E suppresses activity of PRMT1, exhibiting an inhibitory effect on the activation of IκBα and Src, underscoring its potential as a therapeutic agent in inflammatory conditions (101). The therapeutic potential of TC-E 5003 in oncology is demonstrated. Its inhibitory effects on a range of cancer cell lines, including A549, H1299, MCF-7, and MDA-MB-231, are noteworthy. Specifically, at a concentration of 6.0 μM, TC-E 5003 demonstrates significant inhibition rates for these cell lines: 77.11% for A549, 45.44% for A549-INEI, 80.11% for H1299, 86.77% for MCF-7, and 71.43% for MDA-MB-231. Moreover, this study introduces an innovative injectable system, the injectable NBCA ethyl oleate implant (INEI), which has been shown to enhance the average growth inhibition rate in xenografted human lung cancer cells, thereby augmenting the efficacy of TC-E 5003 as a candidate for broad-spectrum antitumor drug development (102).

A separate investigation has illuminated the role of TC-E 5003 in inducing thermogenic responses in primary murine and human subcutaneous adipocytes (103). TC-E5003 treatment amplifies the expression of *Ucp1* (encoding uncoupling protein1) and *Fgf21* (encoding fibroblast growth factor 21), concurrently activating protein kinase A signaling and lipolysis in primary subcutaneous adipocytes derived from both mice and humans. Intriguingly, this effect appears to be independent of PRMT1 activity because the augmentation in Ucp1 and Fgf21 mRNA expression in WT inguinal white adipose tissue cells treated with TC-E was similar to that in PRMT1 KO inguinal white adipose tissue cells. This study provides a broader insight into the potential therapeutic applications of TC-E 5003 beyond its role as a PRMT1 inhibitor.

## DB75 and K313

DB75 (furamidine) and K313 ((2-(4-((4-carbamimidoylphenyl)amino)phenyl)-1H-indole-6-carboximidamide)) are diamidine structured inhibitors (Table 1), exhibiting IC_50_ values of 9.4 μM and 2.6 μM, respectively against the PRMT1 enzyme (73, 104). Docking studies pinpoint that one of the amidine groups interacts with Glu144 and Glu153 which are essential residues for catalytic activity while the other amidine interacts with Glu129 around the SAM binding pocket which may explain its partial competition with SAM binding. Pi-pi stacking is found with Tyr35, Phe36, and Tyr39 of the YFXY motif as well as hydrophobic interactions with Met146, Met155, and Thr158. K313 is about 5 to 10 fold more potent than DB75 in PRMT1 inhibition. In docking analysis, the binding mode of K313 differs from DB75 where K313 stretches from the adenosine region of the SAM binding pocket and through to the methionine portion of SAM. K313 still interacts with the catalytic glutamate residues 153 and 144 as well as the pi-pi interactions found in DB75 with Tyr35, Phe36, and Tyr39. All the SAR data based on testing various diamidine analogs highlight the absolute requirement of the amidine group in PRMT1 inhibition. If the diamidine moieties were changed to dihydroimidazole rings, there would be a decrease in potency. Other disfavored modifications include the use of a flexible linker or a longer linker within the core of the compound as well as replacing the nitrogen found in the cyclic core with either oxygen or carbon (104). Although generally more potent for PRMT1, certain diamidine compounds show modest inhibition of PRMT5 as well. Especially, it was found that compounds containing bulkier moieties and more hydrophobic cores tend to be more potent for PRMT5 while the opposite is true for PRMT1 (104). In PRMT5 docking interaction, the amidines form electrostatic interactions with Glu435, Glu444, and Asp419. Hydrophobic interactions between the inhibitor and residues Leu315, Leu316, Leu436, Leu437, Met420, and Pro314 are observed. With these comparisons, the authors reasoned that the difference in affinity toward the two enzymes may be due to the difference in the structure of the active pockets as PRMT5 has a larger and more solvent exposed cavity than PRMT1. As a result, the shape and rigidity of the diamidine inhibitors can be altered to further increase their selectivity (73, 105).

DB75 and related diamidine inhibitors have been used as a chemical tool to study PRMT1 function in different biological systems. In H9c2, rat embryonic cardiomyocytes, the inhibition of PRMT1 activity by DB75 at 20 μM aggravated Dox-induced reactive oxygen species (ROS) formation and endoplasmic reticulum (ER) stress, pinpointing that the methylation activity of PRMT1 is critical for the protective function in cardiomyocytes (106). DB75 penetrates the plasma membrane of 293T-cells (human embryonic kidney cell line) and inhibits cellular PRMT1 activity on ALY protein (also known as THO complex subunit 4, or THOC4) methylation, thereby regulating the turnover rate of ALY. Treatment with DB75 (at 20 μM) for 72 h leads to significant inhibition of the growth of several leukemia cells. Intriguingly, cell lines derived from Down’s syndrome patients and MLL-AF9 (a fusion oncogene seen in leukemia cells) patient seems more sensitive (107).

**Figure 4:**
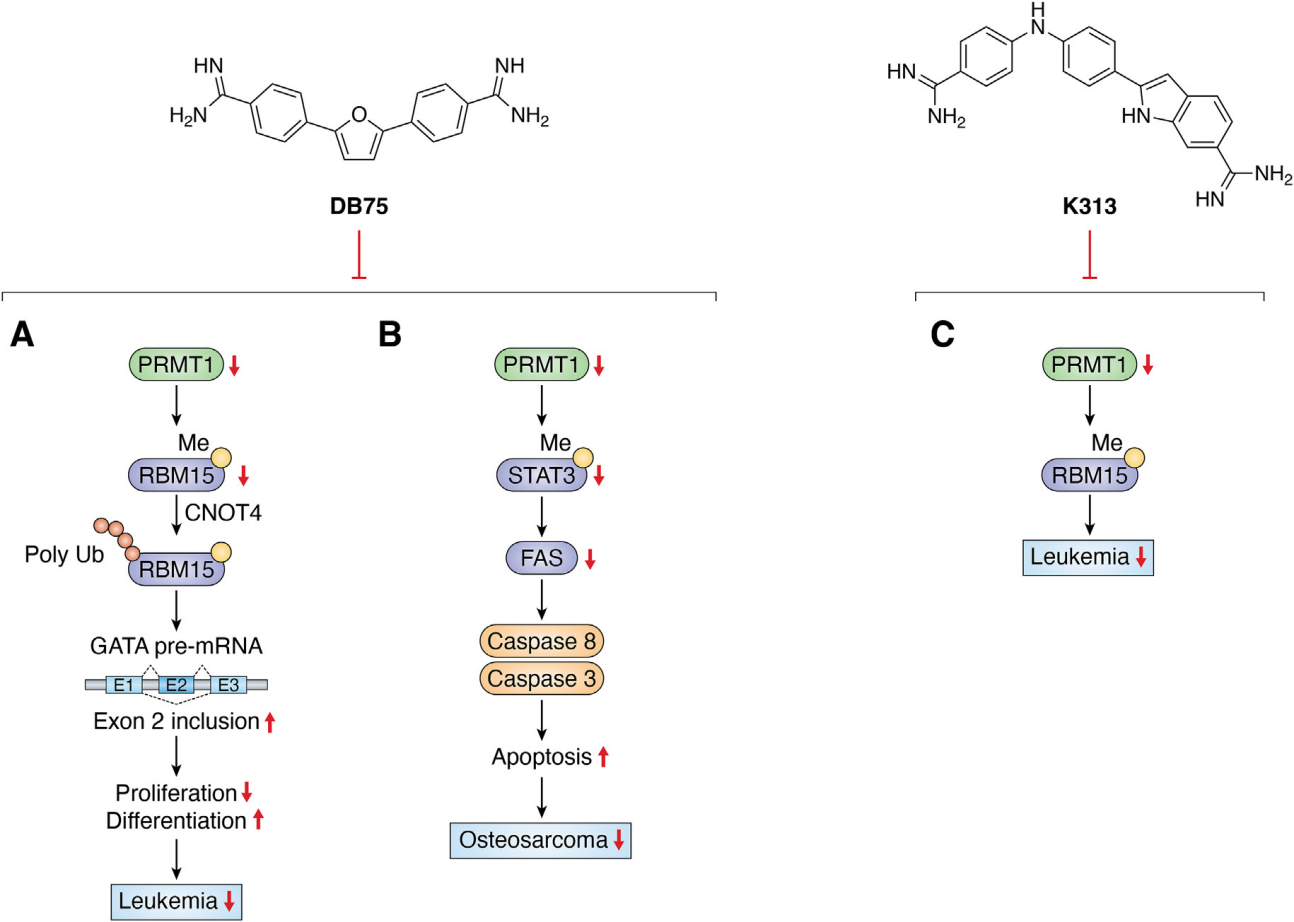
**Biomedical effects of DB75 and K313.***A*, DB75 inhibits PRMT1-mediated methylation of RBM15, leading to its degradation and altering the splicing of key genes involved in megakaryocyte differentiation. *B*, DB75 promotes apoptosis in U-2OS osteosarcoma cells by inhibiting PRMT1, reducing STAT3 arginine methylation, and downregulating FAS transcription. *C*, K313 inhibits MEG01 leukemia cell proliferation by reducing RBM15 methylation, a PRMT1 substrate critical for alternative RNA splicing. PRMT, protein arginine methyltransferase.

A close structural analog of DB75 was used as a tool compound to inhibit PRMT1-mediated Smad6 methylation, revealing that the MTase activity of PRMT1 is essential for bone morphogenetic proteins-induced Smad1/Smad5 phosphorylation and downstream signaling activation (108). DB75 inhibits PRMT1-mediated putative RNA-binding protein 15 (RBM15) methylation at residue R578 (109). Arginine methylation of RBM15 leads to its ubiquitination by the E3 ligase CNOT4 and subsequent degradation. Downregulation of RBM15 changes alternative splicing of the downstream genes such as GATA1 (GATA-binding factor 1), C-MPL (MPL proto-oncogene), and RUNX1 (runt-related transcription factor 1 gene), which are critical for megakaryocyte differentiation. RBM15 binds to the SF3B1-containing complex in methylation-dependent manner. SF3B1 has been shown to be mutated in the myelodysplastic syndrome and other forms of leukemia. Thus, PRMT1-mediated methylation of RBM15 directly links the PRMT to RNA splicing pathways (Fig. 4*A*). Interestingly, Siboni *et al.* (110) found that DB75 impacts on RNA splicing in a myotonic dystrophy type 1 model with equal efficacy and low toxicity compared with another diamidine compound pentamidine. Given our observation that DB75 modulates PRMT1-mediated RBM15-SF3B1 splicing regulators, it could be possible that DB75 also regulates RNA splicing in myotonic dystrophy type 1 through PRMT1 inhibition. This hypothesis has to await future experimental verification.

Samuel *et al.* (111) tested several PRMT1 inhibitors in U87-MG glioblastoma (GBM) cells including DB75 and MS023. DB75 shows a dose-dependent reduction in cell viability of GBM under standard 2D cell culture conditions as well as a spheroid 3D model. As to effect on arginine methylation, molecular phenotype of DB75 is slightly different from that of MS023. Incubation of U87-MG cells with DB75 led to loss of ADMA of a protein next to the 95 kDa marker, while the intensity of the band at 55 kDa increased. This could be because DB75 has relatively better selectivity than MS023 which is a broad inhibitor against multiple type-I PRMTs (PRMT1, 3, 4, 6, 8).

DB75 notably inhibits cell proliferation of 10 different leukemia cell lines at 20 μM (73). A research has illuminated the potential of DB75 as a promising therapeutic agent in osteosarcoma (OS) treatment through its mechanism of inhibiting PRMT1 (112).This inhibition leads to a reduction in STAT3 arginine methylation and activation, which consequently downregulates the transcription of FAS, a key proapoptotic gene, thus promoting apoptosis in U-2OS cells. These findings suggest that targeting PRMT1 could offer a novel and effective approach to managing OS (Fig. 4*B*). Similarly, K313 significantly inhibits the proliferation of leukemia cancer cell lines, concurrently effectuating a reduction in cellular ADMA levels, with a notable IC_50_ of 1 μM in MEG01 cells. In particular, the inhibitory effect of K313 on MEG01 cells is linked to its action in diminishing the methylation status of RBM15, a known substrate of PRMT1 that is pivotal in modulating alternative RNA splicing within the context of leukemia (Fig. 4*C*) (104).

PRMT1 is important for medulloblastoma cell proliferation and survival. As such, it is unsurprising that DB75 and other diamidine compounds significantly reduced cell growth in human medulloblastoma cells (48). Furthermore, PRMT1-mediated survival pathway is critical in neuroblastoma. High PRMT1 expression levels are correlated with poor prognosis of neuroblastoma. The inhibitor K313 treatment decreased the ADMA levels in neuroblastoma SK-N-MC cell line. Pharmacological treatment with diamidine inhibitors exhibited antineuroblastoma efficacy in both cell and animal models (113, 114).

## AZ-PRMT5i-1 and MRTX1719

MTAP deficiency frequently occurs in cancers due to its codeletion with the tumor suppressor gene CDKN2A. The loss of MTAP leads to an accumulation of methylthioadenosine (MTA), which inhibits the enzymatic activity of PRMT5. This creates a metabolic vulnerability in MTAP-deficient cancers, as the inhibition of PRMT5 is more sensitized than normal cells for disrupting essential methylation-dependent processes required for cell survival. As a result, MTAP-deficient cells become selectively dependent on PRMT5 activity, making PRMT5 an attractive therapeutic target. Inhibition of PRMT5 in MTAP-deficient cancers offers a promising strategy to exploit this synthetic lethality, potentially improving outcomes in tumors harboring this common genomic alteration (115, 116, 117).

MTA-cooperative PRMT5 inhibitors are designed to selectively target the PRMT5 form in MTAP-deficient cancer cells by enhancing the natural inhibitory effect of accumulated MTA, sparing normal tissues. This selective inhibition strategy has become a promising approach used by pharmaceutical companies in cancer drug discovery, as it has the potential to improve the therapeutic index and reduces toxicity. In a recent example, the MTA-cooperative PRMT5 inhibitor, AZ-PRMT5i-1, has shown potent inhibitory effects on specific cancer cell lines, particularly those with MTAP deficiency. In preclinical studies, AZ-PRMT5i-1 demonstrated significant antiproliferative activity in MTAP-deficient HCT-116 colon cancer cells. The IC_50_ for AZ-PRMT5i-1 in these MTAP KO HCT-116 cells was 660 nM, while in the MTAP WT counterpart, the IC_50_ is 4.4 μM, showing over a 6-fold selectivity for the MTAP-deficient cells. This selectivity highlights the drug's potential for specifically targeting MTAP-deleted tumors, minimizing off-target effects in MTAP-proficient tissues (118).

In another example, the MTA-cooperative PRMT5 inhibitor, MRTX1719, demonstrates significant inhibitory effects on cancer cells with MTAP deletions by selectively binding to the PRMT5/MTA complex. In preclinical tests, MRTX1719 showed robust antiproliferative activity in MTAP-deleted HCT116 colon cancer cells, with an IC_50_ of 8 nM, compared to an IC_50_ of 653 nM in WT HCT116 cells, highlighting its over 70-fold selectivity for MTAP-deficient cells. Additionally, in LU99 lung cancer xenograft models, MRTX1719 exhibited dose-dependent tumor growth inhibition and nearly complete inhibition of PRMT5 activity at well-tolerated doses. This inhibitor shows promise as a targeted therapy for MTAP-deficient cancers, including nonsmall cell lung cancer, mesothelioma, and pancreatic cancer, with early signs of clinical efficacy in these cancer types (119).

## EPZ015666 and GSK591

**Figure 5:**
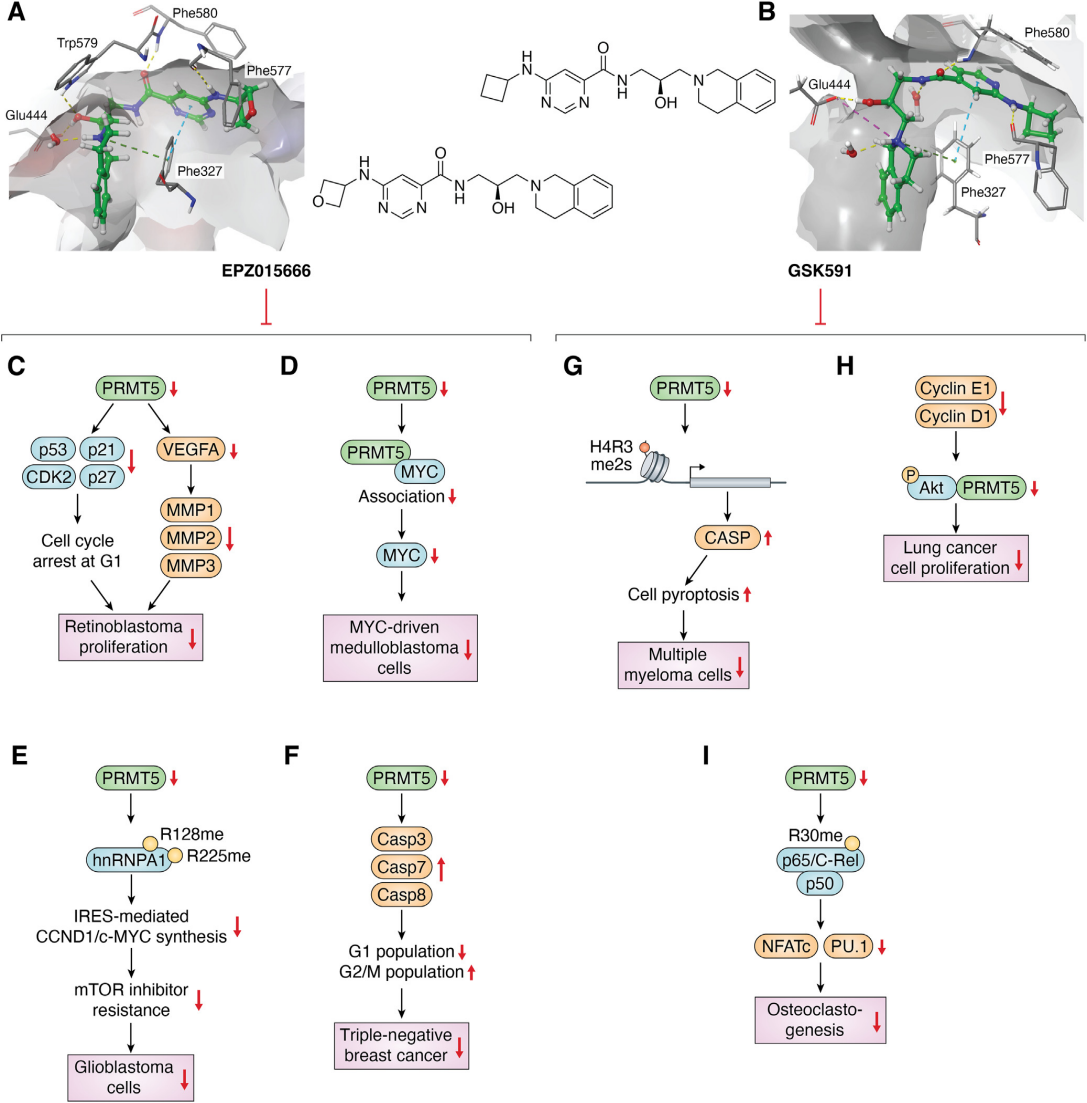
**Biomedical effects of EPZ015666 and GSK591.** EPZ015666, through the inhibition of PRMT5 activity, downregulates the proliferation of diverse cancer cell types, including retinoblastoma cells, MYC-driven medulloblastoma cells, glioblastoma cells, and triple-negative breast cancer cells. The inhibitory effect of GSK591, which has similar structure of EPZ015666, on lung cancer cells, multiple myeloma (MM) cells and osteoclastogenesis. *A*, EPZ015666 in PRMT5 (PDB: 4X61). *Green structure*: EPZ015666; *yellow dashes*: hydrogen bonds; *light blue dashes*: pi-pi interactions; *green dashes*: pi-cation interactions. *B*, GSK591 in PRMT5 (PDB: 5C9Z). *Green structure*: GSK591; *pink structure*: SAH; *yellow dashes*: hydrogen bonds; *pink dashes*: ionic interactions; *light blue dashes*: pi-pi interactions; *green dashes*: pi-cation interactions. *C*, EPZ015666 inhibits the growth and proliferation of retinoblastoma se(RB) cells by regulating the expression level of cell cycle-related proteins, including P53, P21, P27, and CDK2, leading to cell cycle arrest at the G1 phase. These data suggest that EPZ015666 is a potential strategy for treatment of RB. EPZ0156666 also inhibits the carcinogenesis of RB by regulating MMP proteins. *D*, EPZ015666 targets MYC-driven medulloblastoma by downregulating the association of PRMT5 and Myc. *E*, EPZ015666 disrupts IRES activities of cyclin D1 and c-MYC, enhancing the sensitivity of GBM cells to mTOR inhibitors and improving treatment efficacy, both alone and in combination with PP242 or *via* extracellular vesicles. *F*, EPZ015666 reduced TNBC tumor growth through apoptosis and G2/M cell cycle arrest, driven by increased levels of caspase 3, 7, and 8. *G*, GSK591 impedes PRMT5 activity, elevating CASP1 expression in MM cells through the diminution of H4R3me2s levels at the CASP1 promoter. *H*, GSK591 inhibits lung cancer cell growth by inhibiting PRMT5, leading to decreased phosphorylation of key residues and lower levels of cell cycle proteins cyclin E1 and cyclin D1 *via* the Akt/GSK3β pathway. *I*, GSK591 inhibits PRMT5 to decrease the methylation level of R30 on p65 and reduce NFATc1 and PU.1 levels. GBM, glioblastoma; IRES, internal ribosome entry site; MMP, matrix metalloproteinase; mTOR, mechanistic target of rapamycin; MYC, myelocytomatosis oncogene; PRMT, protein arginine methyltransferase; RB, retinoblastoma; SAH, S-adenosylhomocysteine; TNBC, triple-negative breast cancer.

EPZ015666 has emerged as a highly potent inhibitor of PRMT5 with an *in vivo* IC_50_ of 22 nM (120). In the crystal structure with PRMT5, EPZ015666 is shown to interact with key residues involved in the catalysis of the methyl transfer (Fig. 5*A*). The amide of Phe580, side chain of Glu444, and the side chain of Glu345 through a water-mediated interaction bind with the tertiary nitrogen in the THIQ ring. Of particular note, a pi-pi interaction is found between Phe327 and the THIQ ring. Some other interactions found include a pi-cation interaction between the aryl secondary nitrogen and Tyr312 and Phe588 as well as hydrogen bonding between Ser447 and the amide of EPZ015666.

The administration of EPZ015666 to mantle cell lymphoma cell lines markedly suppresses SmD3 methylation (120). This inhibition curtails cellular proliferation and induces cell death within the nanomolar range. In a mantle cell lymphoma xenograft model, EPZ015666 demonstrates notable antitumor efficacy in a dose-dependent manner, further solidifying its therapeutic potential in cancer treatment. PRMT5 plays important roles in promoting retinoblastoma (RB) development (121). It has been demonstrated that EPZ015666 effectively inhibits the growth and proliferation of RB cells by regulating the expression level of cell cycle-related proteins, including p53, p21, p27, and CDK2, leading to cell cycle arrest at the G1 phase (Fig. 5*C*). These data suggest that EPZ015666 is a potential strategy for treatment of RB. Moreover, Jiang *et al.* (122) found that EPZ0156666 inhibits the carcinogenesis of RB by regulating matrix metalloproteinases (MMPs). Mechanistically, silencing of PRMT5 reduces H3K4me3-mediated vascular endothelial growth factor A (VEGFA) transcription and further suppresses the expression of MMP1, MMP2, and MMP9 to inhibit the proliferation of RB.

An aberrant increase in PRMT5 level was detected in myelocytomatosis oncogene (MYC)-driven medulloblastoma cells (123). Treatment with EPZ015666 resulted in downregulation of both PRMT5 and MYC protein expression, suppression of cell growth, and induction of apoptosis in MYC-driven medulloblastoma cells associated with G1/S cell cycle arrest (Fig. 5*D*). It is important to note that this treatment is specific for MYC-driven medulloblastoma but not non-MYC-amplified medulloblastoma, as PRMT5 physically interacts with and regulates MYC protein expression.

The therapeutic potential of EPZ015666 extends to the treatment of glioblastoma, a notably aggressive brain tumor (124). The pivotal study showed that EPZ015666 effectively disrupts GBM cell cycle progression by accumulating cells in the G2/M phase and preventing their entry into the S-phase. This action significantly suppresses the proliferation of GBM cells, both *in vitro* and *in vivo*. The underlying mechanism is attributed to the inhibition of PRMT5, which modulates the splicing of detained introns in genes that are crucial for GBM cell proliferation. This insight into the molecular action of EPZ015666 underscores its potential as a novel therapeutic strategy in combating GBM. Furthermore, the research conducted by Gao *et al.* (125) elucidated that EPZ015666 impairs the internal ribosome entry site activities of cyclin D1 and c-MYC by inhibiting the methylation of hnRNP A1. This inhibition is particularly significant as the induction of internal ribosome entry site activity is a primary mechanism underlying the drug resistance observed with mechanistic target of rapamycin (mTOR) inhibitors. Consequently, EPZ015666 effectively sensitizes GBM cells to mTOR inhibitors. This sensitization has been demonstrated in both *in vitro* studies and xenograft mouse models, where EPZ015666, in combination with the mTOR inhibitor PP242, exhibited synergistic anti-GBM effects (Fig. 5*E*). Additionally, a recent report shows that small extracellular vesicles loaded with EPZ015666 significantly enhance the compound's efficacy in inhibiting GBM proliferation, as compared to the use of EPZ015666 alone (126). This novel delivery method underscores the potential for improved therapeutic strategies in GBM treatment.

In a study focused on TNBC, EPZ015666 was evaluated in xenograft murine models, demonstrating a notable 39% reduction in tumor growth compared to untreated controls. This inhibitor effectively decreases the proliferation and viability of TNBC cells by facilitating apoptosis and inducing G2/M cell cycle arrest. Specifically, EPZ015666 upregulates the expression of caspase 3, caspase 7, and caspase 8, thereby impeding tumor progression. Furthermore, when combined with erlotinib, an epidermal growth factor receptor (EGFR) inhibitor, EPZ015666 exhibits additive anticancer effects on MDA-MB-468, BT20, and HCC70 breast cancer cell lines, enhancing the efficacy of either treatment compared to when they were used alone (Fig. 5*F*) (127, 128).

EPZ015666 possesses potent inhibitory properties against CRC harboring mutations in the KRAS gene, a mutation prevalent in approximately 45% of all CRC cases (129). The efficacy of EPZ015666 in inhibiting PRMT5 was evaluated across both KRAS-mutant and WT CRC cell lines. The findings revealed a significantly heightened efficacy in KRAS-mutant cell lines, characterized by improved cell survival inhibition and an increased incidence of G2 phase cell arrest, compared to nonmutant cell lines (129). Furthermore, because PRMT5 has been demonstrated to play crucial roles in leukemia and lymphoma treatment, administration of EPZ015666 caused apoptosis in adult T-cell leukemia/lymphoma cell lines. It improves survival outcomes and decreases tumor burden of human T-cell leukemia virus type 1 infected humanized immune system mice and xenograft mice (130).

GSK591 (also known as EPZ015866 or GSK3203591) is a close structural analog of EPZ015666. It demonstrates exceptional potency *in vitro* with an IC_50_ of 4 nM against PRMT5 (Fig. 5*B*) (131). Therefore, similar as EPZ015666, it is a valuable tool for *in vitro* biochemical assay to explore the inhibitory impact of PRMT5. Xia *et al.* (132) have elucidated that GSK591 enhances the expression of CASP1 in multiple myeloma (MM) cells by diminishing the level of H4R3me2s at the CASP1 promoter, thereby inducing pyroptosis in these cells. Intriguingly, the suppression of PRMT5 expression was observed to reverse both the phenotypic manifestations and the expression of key markers, such as N-GSDMD, IL-1b, and IL-18 (Fig. 5*G*). Given the correlation between elevated PRMT5 expression, reduced CASP1 levels, and poorer clinical outcomes in MM patients, the therapeutic application of GSK591 presents a promising avenue for treatment. This insight offers a potential strategy to improve clinical interventions for MM, targeting the molecular interplay between PRMT5 and CASP1. Additionally, a recent investigation found that AML cell lines harboring mutations in splicing factors, including SF3B1, SRSF2, and U2AF1, show heightened sensitivity to GSK591 (133). *In vitro* studies have further substantiated the efficacy of GSK591 in impeding the proliferation of human MLL-AF9-rearranged AML cell lines and patient-derived samples. Importantly, a synergistic effect was observed when combined with type I PRMT inhibitors or SF3B inhibitors. For instance, the concomitant use of MS023 and GSK591 significantly extended survival in recipient mice engrafted with both Srsf2^WT^ and Srsf2^P95H^ MLL-AF9 leukemia cells. This synergy highlights the therapeutic potential of targeting both PRMTs and splicing factor mutations in AML. Ding *et al.* (134) have demonstrated the inhibitory effects of both GSK591 and EPZ015666 on osteoclastogenesis. Through *in vitro* assays, GSK591 was found to be more effective than EPZ015666 in hindering RANKL-induced osteoclast differentiation. Mechanistically, GSK591 disrupts the formation of actin rings, an essential component for the attachment of mature osteoclasts to the bone surface. This disruption of actin ring formation subsequently leads to a reduction in bone resorption. Additionally, GSK591 was observed to significantly suppress the expression levels of NFATc1 and PU.1, which are key transcriptional activators in the process of osteoclast differentiation. These findings highlight the potential of GSK591 as a therapeutic agent in conditions characterized by excessive osteoclast activity (Fig. 5*I*).

A recent research has underscored PRMT5 as a promising therapeutic target in GBM (135). GSK591 has demonstrated significant efficacy in suppressing the growth and proliferation of GBM cells, with effective concentration (EC_50_) values in the low micromolar range. The inhibition of PRMT5 by GSK591 triggers a spectrum of cellular responses, including senescence, apoptosis, and disruption of transcriptome alternative splicing. Parallel studies have shown that GSK591 also impedes the growth and proliferation of lung cancer cells. Zhang *et al.* (59) reported that GSK591-mediated PRMT5 inhibition results in diminished phosphorylation of Thr308 and Ser473 residues of Akt. This inhibition subsequently leads to a reduction in the expression of key cell cycle proteins, namely cyclin E1 and cyclin D1, which are downstream targets of the Akt/GSK3β pathway. Corroborating these findings, Li *et al.* observed that GSK591 treatment reduces cell viability and induces apoptosis in lung cancer cells (Fig. 5*H*) (136). Furthermore, another study revealed that PRMT5 inhibition by GSK591 hampers the epithelial-mesenchymal transition in lung cancer cells by modulating the EGFR/Akt signaling pathway (137).

## LLY-283

**Figure 6:**
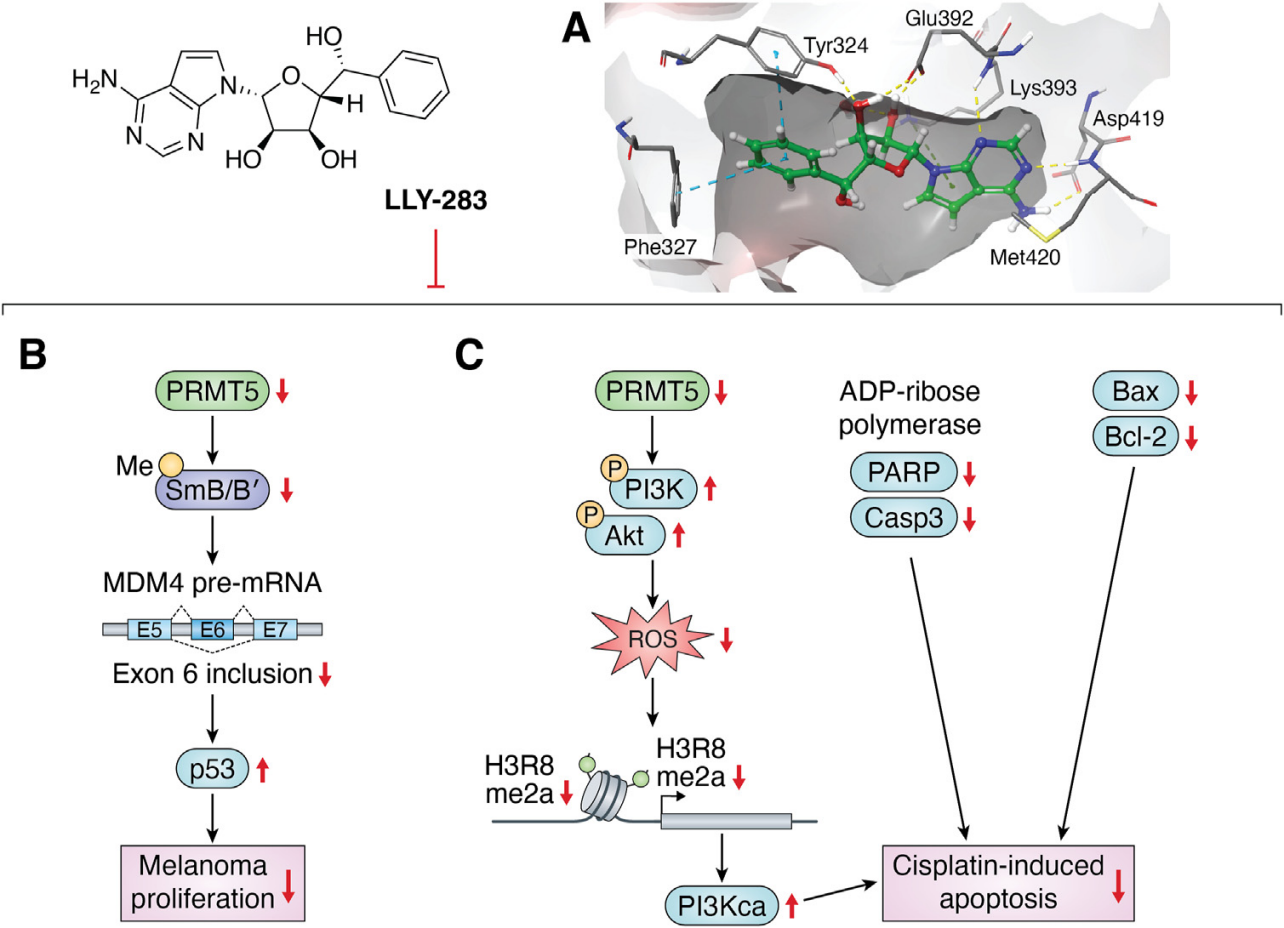
**The inhibitory effect of LLY-283 on melanoma cells proliferation and cisplatin-induced apoptosis.***A*, the interaction of LLY-283 in PRMT5 (PDB:6CKC). *Yellow dashes*: hydrogen bonds; *light blue dashes*: pi-pi interactions; *green dashes*: pi-cation interactions. *B*, LLY-283 shows antiproliferative effects on various cancers by blocking SmB/B′ protein methylation, affecting MDM4 splicing, reducing full-length MDM4 levels, and causing cell cycle arrest and apoptosis in melanoma cells. *C*, LLY-283 mitigates cisplatin-induced ototoxicity by inhibiting apoptosis pathways, notably through the reduction of ROS levels and modulation of the Bax/Bcl-2, PARP, and caspase 3 signaling pathways. It also enhances spiral ganglion neuron viability *via* the PI3K/AKT pathway, indicating potential for treating noise-induced hearing loss. PRMT, protein arginine methyltransferase; PDB, Protein Data Bank; PARP, poly-ribose polymerase; ROS, reactive oxygen species.

LLY-283 has emerged as a highly potent and selective PRMT5 inhibitor, exhibiting remarkable efficacy both *in vitro* and *in vivo* (138). It inhibits PRMT5 enzymatic activity with an IC_50_ of 22 ± 31 nM in biochemical assays and 25 ± 1 nM for MCF7 cellular assays. Contrasting with substrate competitive inhibitors like EPZ015666 and GSK591, LLY-283 operates as a cofactor-competitive inhibitor, binding directly to the SAM pocket (Fig. 6*A*). As such, it shares similar interactions found with other analogs of the cosubstrate. The side chain of Asp419 and the amide of Met420 form hydrogen bonds with the adenine ring. As well, Tyr324 forms hydrogen bonds with the hydroxyls of the ribose ring. Of note, Phe327 appears to be displaced by the phenyl ring of LLY-283. It was shown that converting the S-centered hydroxyl to an R center for LLY-284 yields a much lower potency (IC_50_ = 1074 ± 53 nM). Antiproliferative effects of LLY-283 were demonstrated across a spectrum of cancer cell lines, including breast, gastric, lung cancers, and hematological malignancies (138). Notably, LLY-283 impedes the methylation of SmB/B′ proteins, consequently altering the splicing of MDM4 regulator of p53 (MDM4) (138). This alteration leads to reduced levels of full-length MDM4, resulting in cell cycle arrest and apoptosis in A375 human melanoma cell line (Fig. 6*B*). These findings underscore LLY-283's considerable potential as a versatile cancer therapeutic and as an instrumental probe molecule for elucidating PRMT5's biological role in oncogenesis.

LLY-283 is shown as a promising therapeutic agent for GBM (135). In preclinical GBM models, oral administration of LLY-283 has shown significant survival benefits. Crucially, LLY-283 is capable of crossing the blood-brain barrier (BBB). BBB presents a critical challenge in the development of GBM treatments, as many potential therapies fail clinical trials due to their inability to effectively penetrate the BBB. Therefore, LLY-283 not only offers therapeutic potential for GBM but also sets a precedent in overcoming a major hurdle in drug delivery to the central nervous system. Furthermore, Chen *et al.* (139) demonstrated that LLY-283 inhibited the proliferation and metastasis of head and neck squamous cell carcinoma by suppressing expression of PRMT5 and Ki-67. Recent investigations have delved into the potential synergies of combination therapies involving LLY-283 (140). One study has demonstrated that LLY-283 effectively reduces the level of SDMA in SmB proteins, thereby significantly augmenting the inhibitory effects of tyrosine kinase inhibitors (TKIs) like gilteritinib and quizartinib on FLT3-ITD positive MOLM-13 and MV4-11 AML cells. This finding underscores the therapeutic promise of coupling a PRMT5 inhibitor such as LLY-283 with a FLT3 TKI, potentially offering a more effective strategy to target and eradicate FLT3-ITD AML stem cells.

Beyond its cancer-inhibiting properties, LLY-283 has also been shown to exert a protective effect against cisplatin-induced ototoxicity in auditory cells (141). This protective role may be attributed to its ability to inhibit cochlear cell apoptosis, which is often precipitated by oxidative stress. Notably, in cisplatin-exposed HEI-OC1 cells, treatment with LLY-283 significantly reduces the levels of ROS, suggesting its potential as a therapeutic agent in mitigating the ototoxic side effects commonly associated with cisplatin chemotherapy (Fig. 6*C*). At the same time, it has been shown that Bax/Bcl-2, PARP, and Caspase 3 are lower with the treatment of LLY-283 which promote the protective effect. Likewise, LLY-283 has shown potential in neuroprotective applications, particularly in the auditory system (142). By mitigating ROS accumulation and modulating the PI3K/AKT pathway, LLY-283 treatment has been observed to increase the viability of spiral ganglion neurons. These findings suggest that LLY-283 could be a viable therapeutic candidate for addressing noise-induced hearing loss. However, more work is required to fully elucidate the underlying mechanisms of LLY-283's protective effects against noise-induced hearing loss, paving the way for its potential clinical application in auditory protection.

Furthermore, LLY-283 has demonstrated promising neuroprotective effects against cerebral ischemia/reperfusion injury, primarily through the suppression of the NF-κB/NLRP3 axis (143). Administration of LLY-283 results in a marked reduction in cerebral inflammation and neural pyroptosis, as well as a notable attenuation of the brain infarct region. as such, LLY-283 contributes to significant improvements in neurological function. These findings collectively suggest that targeting PRMT5 with LLY-283 could represent a novel and effective therapeutic strategy for the treatment of cerebral ischemia/reperfusion injury.

## SGC707

SGC707 emerges as a potent inhibitor of PRMT3, distinguished by its remarkable allosteric inhibitory properties, as evidenced by an IC_50_ of 31  ± 2 nM and a K_D_ of 53  ± 2 nM with notable selectivity (74). SGC707 was an optimized inhibitor based on a lead compound which contains a dihydro-thiadiazole and a cyclohexene ring. An analog was synthesized alongside SGC707 in which a naphthalene ring replaced the isoquinoline. This change in the bicyclic moiety rendered the compound inactive with concentrations up to 100 mmol. In the crystal structure, Thr466 was found to hydrogen bond with the isoquinoline as well as Arg396 with the carbonyl. The amide was found to either have direct or water-mediated interactions with Lys392 and in addition, Glu422 formed bonds with the nitrogen of the urea.

Crucially, SGC707 has demonstrated the capacity to interact effectively with PRMT3, significantly repressing its MTase activity in cellular environment (74). Furthermore, its favorable bioavailability profiles render SGC707 well-suited for *in vivo* animal studies. Recent research has shed light on the therapeutic potential of SGC707-mediated PRMT3 inhibition, particularly in the context of hepatic disorders (144). It has shown promising effects in attenuating hepatic steatosis, reducing plasma lipid levels, and diminishing atherosclerosis susceptibility. Another study demonstrates PRMT3 facilitates the growth HCC by promoting glycolysis through the enhancement of arginine methylation of lactate dehydrogenase A (145). It suggests that PRMT3 acts as an oncogenic factor in HCC, and targeting PRMT3 with its inhibitor, SGC707, effectively diminishes PRMT3-induced glycolysis and tumor growth, presenting a novel therapeutic approach for HCC treatment. These findings underscore the potential of pharmacological PRMT3 inhibition, particularly with SGC707, as a viable therapeutic strategy for conditions such as nonalcoholic fatty liver disease and dyslipidemia/atherosclerosis. Moreover, it has been elucidated that PRMT3, through the methylation of hypoxia-inducible factor 1 alpha (HIF1α) at the R282 site, stabilizes HIF1α, thereby enhancing its oncogenic activity and promoting angiogenesis (146). Inhibition of PRMT3 by SGC707 disrupts this methylation process, leading to decreased stability and activity of HIF1α, which in turn attenuates tumor angiogenesis and growth. This mechanistic insight positions SGC707 as a promising therapeutic agent for CRC treatment, especially in tumors exhibiting upregulated PRMT3 activity and resistance to traditional antiangiogenic therapies.

Moreover, a study demonstrates that SGC707 targets PRMT3, a key regulator in mesenchymal stem cell osteogenesis, leading to the attenuation of bone remodeling and the induction of an osteopenia phenotype in mice (147). By inhibiting PRMT3, SGC707 disrupts the enzyme's role in promoting miR-3648 expression through the enhancement of histone H4 arginine 3 asymmetric dimethylation (H4R3me2a) at the gene's promoter region, highlighting the potential of SGC707 as a therapeutic agent for bone regeneration and osteopenia disorders.

## EZM2302, TP064, and MS049

EZM2302 is a potent therapeutic agent for CARM1 by competitively binding to the peptide-binding pocket (148). From a former compound, the 1-amino-3-phenoxylpropan-2-ol substituent was found to be of great importance for binding and was carried over to EZM2302 for optimization. For EZM2302, it was observed in the crystal structure that this substituent when methylated at the terminal amine hydrogen bonds with Glu266. The aromatic ring that this substituent is bound to is shown to undergo pi-pi stacking with Phe152, Tyr261, and Phe474 as is seen in the precursor compound as well. Further optimization of the aromatic rings lead to the use of a dimethylisoxazole which provides additional van der Waals interactions with Glu192, Tyr261, and Lys470 with the 2,7-diazaspiro[3.5]nonyl group forming van der Waals interactions with Phe474 as well. It can be further stabilized by the presence of S-adenosylhomocysteine.

*In vitro* studies have demonstrated that EZM2302 displays antitumor activity against multiple myeloma, with day 14 IC_50_ value less than 100 nM in various cell lines. Notably, the compound's mode of action appears to be primarily cytostatic rather than cytotoxic, as evidenced by the inhibition of cellular proliferation without inducing significant cell death in these cell lines (148). Beyond multiple myeloma, preclinical investigations have expanded to include a spectrum of malignancies, such as AML, diffuse large B-cell lymphoma, and gastric cancer (149, 150, 151). These studies indicate that EZM2302 has a broad range of potential applications in oncology, making it a promising candidate for further clinical development.

TP-064 is another potent, selective, and cell-active chemical probe of CARM1. As seen in the cocrystal complex, the terminal phenyl ring formed pi-pi interactions with Pro473 and Phe475 (152). In addition to this, the pyridinyl group provided pi-pi stacking interactions with Phe153 as well. The oxygen of the diphenyl ether portion of the structure was observed to have hydrogen bonding with Asn266 while the tertiary amide of TP-064 formed water-mediated hydrogen bonding with Ser146 and Lys471. The authors stated that the specificity of this compound with regard to other type 1 PRMTs may be due to the loss of certain interactions such as the loss of the pyridinyl pi-pi interaction when TP-064 is docked in PRMT6. Also, the loss of both the pyridinyl pi-pi interaction and the diphenyl ether hydrogen bond in the PRMT3 model may rationalize the drop in potency TP-064 experiences when inhibiting PRMT3. Only PRMT4 was observed to maintain additional interactions with Ser146, Lys471, Pro473, and Phe475 which may explain TP-064’s potency regarding this enzyme.

TP-064 inhibited the MTase activity of CARM1 with IC_50_ < 10 nM in biochemical assays and TP-064 treatment inhibited the proliferation of a subset of MM cell lines (152). The mechanism is that inhibition of PRMT4 by TP-064 treatment reduced the proportion of NCI-H929 cells in S and G2/M phases while increasing the G1 phase fraction. Additionally, PRMT4 was found to express high in white blood cells of atherosclerotic patients (153). The study showed that TP-064 treatment decreases LPS-activated tumor necrosis factor-alpha secretion by blood monocytes, even in the context of higher neutrophil counts. In addition, it has been observed that *in vivo* treatment of TP-064 is able to systemically downregulate PRMT4-dependent signaling pathways, including peroxisome proliferator-activated receptor gamma-regulated lipolysis in white adipose tissue, hepatic glycogen metabolism, and insulin levels and signaling. Therefore, PRMT4 might be a novel therapeutic target in obesity.

MS049 is a dual inhibitor of CARM1 and PRMT6. The IC_50_ value of MS049 against CARM1 is 34 ± 10 nM and against PRMT6 is 43 ± 7 nM (154). Such high potency is quite impressive given the relatively small size of the compound. In investigating the SAR for this compound, the structure was analyzed in three different portions: the piperidinylethanamine moiety, the middle linker, and the phenyl moiety. With the piperidinylethanamine moiety, it was observed that while various nonbulky substituents on the terminal amine was acceptable though the number of atoms between the terminal amine and the piperidinyl nitrogen largely affected the compound’s potency. With regards to the linker, two atom linkers generally improved potency for both PRMT4 and PRMT6 including a heteroatom in the linker led to polar interactions as well, leading to the final linker in the form of the ether found in the final compound. As for the final portion of MS049, modifications to the phenyl ring provided for little change to the inhibitor’s potency save for three molecules. For simplicity’s sake, the authors opted to maintain the unsubstituted phenyl ring but mentioned that there was room for further optimization in future studies of the compound. MS049 robustly decreased the H4R3me2a level in HEK293T cells and show no toxicity at up to 50 μM. One study indicated that MS049 was able to increase, in a gene-specific manner, the number of active alleles/cells before and after hormonal stimulation (155). This suggests that mechanisms do indeed exist to modulate hormone receptor responses at the single cell and allele level. Moreover, the inhibition of CARM1 by MS049 decreased the migration and invasion abilities of breast cancer cells that expressed lysine-specific demethylase 1 WT, leading to increased ubiquitination and degradation of lysine-specific demethylase 1 (156).

## MS117 and SGC6870

MS117, the first highly effective and functionally irreversible covalent inhibitor of PRMT6, is designed based on the cocrystal structure of PRMT6-MS023 (157). This inhibitor stands out for its exceptional inhibitory potency against PRMT6, with an IC_50_ of 18 ± 2 nM. The mechanism of action of MS117 revolves around its role as a Michael acceptor in which the thiol of Cys50 attacks the terminal acrylamide forming the covalent bond. As this compound is based on MS023 with the isopropoxy substituent being replaced by the acrylamide at the meta position of the aromatic ring, MS117 interacts with PRMT6 in a similar manner. Aside from the interactions that the isopropoxy group provided, the other notable interactions found in MS023 can be found in the cocrystal structure of PRMT6 and MS117 as well. These interactions include the hydrogen bonding observed in the tertiary amine with His317 and the primary amine with Glu155. The water-mediated interactions with the primary amine and residues Glu164 and Met157 were maintained as well. In cellular assays involving MCF-7, PNT2, and HEK293T cell lines, MS117 has been shown to efficaciously diminish H3R2me2a cellular levels, all the while exhibiting no detectable cellular toxicity.

Characterized as an allosteric inhibitor, SGC6870 distinguishes itself with extraordinary selectivity for PRMT6, markedly distinct from other MTases. Exhibiting an IC_50_ of 77 ± 6 nM, SGC6870 underscores its substantial inhibitory prowess against PRMT6 (158). In the crystal structure, the binding of SGC6870 was found to move residues Gly158 through Met166 to accommodate for the probe. The backbone nitrogen of Gly158 forms a hydrogen bond with the diazepine while the exoamide forms a hydrogen bond with the backbone nitrogen of Gly160. Pi-pi interactions form with Tyr159 and the thiophene as well as Trp156 with the dimethylphenyl substituent of the diazepine. It is of note that the R-centered dimethylphenyl was found to be of importance in SAR studies as the inhibitor containing the S-centered substituent displayed a considerable decrease in potency. In addition to the former interaction, residues Leu267, Val271, and Pro345 have hydrophobic interactions with the methylphenyl substituent of the diazepine. Crucially, both MS117 and SGC6870 have demonstrated significant inhibition of PRMT6 activity in cellular contexts, maintaining a nontoxic profile. These inhibitors emerge as instrumental tools, paving the way for in-depth exploration of PRMT6's roles in both physiological and pathophysiological contexts.

## SGC3027

SGC3027 is the prodrug form of SGC8158, a selective inhibitor of PRMT7. Releasing the active component depends on the intracellular metabolism by reductases (159). It stands as the first potent, selective, and cell-active chemical probe for PRMT7. This compound uses a prodrug strategy coined as trimethyl lock which takes advantage of intracellular metabolism to release the target molecule. Through metabolism *via* reductases, the ketones convert into phenolic alcohols which is the trigger for release of the active compound. The steric hindrance of the methyl groups allow for the optimal configuration of the hydroxyl to react with the labile linker to form an ester and thus breaking the bond between the prodrug moiety and the active drug. With regards to the metabolite’s binding, SGC8158 was crystalized with PRMT7 from *Mus musculus* which has a 93% alignment with human PRMT7, and importantly, has the binding pockets of both SAM as well as substrate conserved. The ribose ring in the inhibitor aligns quite well with the protein cocrystallized with S-adenosylhomocysteine while the two phenyl rings work to displace Trp314 of the THW loop to form a pi-pi interaction with Trp282. Despite the displacement of parts of the THW region, binding of the substrate peptide did not seem to be affected hinting that the specificity of this inhibitor may be due to its interaction with this loop.

In cellular assays using C2C12 cells, SGC3027 exhibits notable efficacy in inhibiting the methylation of HSP70, with an IC_50_ of 1.3 μM (160). The research has revealed that SGC3027-mediated inhibition of PRMT7 can impede spermatogonial proliferation by downregulating the PRMT7/AKT3 pathway (160). Moreover, apigenin, a prevalent dietary flavonoid with recognized anticancer properties, has been identified as an effector of PRMT7 in myoblasts, playing a vital role in male germ cell development. The administration of SGC3027 has been shown to reduce PRMT7 protein expression and AKT phosphorylation effectively (161). Additionally, it decreases the protein levels of cell cycle regulators CCNA2 and CDK2 in spermatogonia treated with 5 μM SGC3027. Interestingly, this reduction can be partially reversed by supplementing with 10 μM SC79, a specific AKT activator, thereby elucidating the intricacies of the PRMT7/AKT pathway in cellular proliferation and development.

## Summary and perspective

In this essay, we have reviewed the key tested preclinical manifestations of some of the most important PRMT inhibitors developed so far, highlighting their considerable potential in therapeutic intervention of various biomedical conditions, most notably in the realm of cancer treatment. Our discussions encompass a wide spectrum of PRMT inhibitors, outlining how they act on PRMT enzymes, their effectiveness in combating diverse forms of cancer, and their potential applications in treating other illnesses. The extensive applications of PRMT inhibitors to date either in cell lines or animal models, such as DB75, MS023, EPZ015666, GSK591, and LLY-283, underscores their significant values in addressing a wide spectrum of disease challenges, especially as anticancer drugs. These inhibitors exhibit remarkable efficacy in various cancer types including multiple myeloma, acute myeloid leukemia, breast cancer, and glioblastoma. Their mechanisms of action span from substrate competitive to cofactor-competitive to allosteric binding, demonstrating the broad spectrum of therapeutic strategies. Beyond their application in oncology, PRMT inhibitors also exhibit remarkable versatility and potential therapeutic effects in other biomedical contexts. Particularly, the exploration of PRMT inhibitors in neurodegenerative and cardiovascular disorders suggests their broader therapeutic scopes. Looking forward, the field of PRMT inhibitor development presents a number of promising avenues and opportunities. Firstly, the design of combination therapies, where PRMT inhibitors are used in conjunction with other cancer therapeutics such as TKIs and EGFR inhibitors, could enhance treatment efficacy and overcome drug resistance. Also, some type I PRMT inhibitors could be combined with type II PRMT inhibitors to enhance their therapeutic strengths in synergistic ways. Secondly, exploring the potential of PRMT inhibitors in other disease types could generate novel therapeutic opportunities. For example, combining with immune checkpoint inhibitors, PRMT inhibition has emerged as a promising therapeutic event for immunotherapy, which demands more efforts to unravel the underlying molecular mechanisms to facilitate further antitumor drug development (162, 163, 164). Another exciting prospect lies in the refinement of delivery systems, such as encapsulating PRMT inhibitors in extracellular vesicles, to enhance drug efficacy and reduce systemic side effects (126). Moreover, although there have been studies to investigate the functions of PRMTs in critical cellular cascades such as regulation of gene expression, processing of precursor mRNA, repair of DNA damage, the body's immune response to cancer, and the defense against viral infection, a systemic and integrated investigation of functional roles of PRMTs in regulating total biological processes is important for the field to comprehend pharmacogenomics of PRMT inhibition at the omics level and develop further novel therapeutic modalities (164, 165). Additionally, several studies have demonstrated the interactions between different PRMT family members and their biological effects, suggesting the potential for combination therapies using two PRMT inhibitors (166, 167, 168). Such a combination could potentially enhance therapeutic efficacy by targeting multiple pathways simultaneously or by overcoming compensatory mechanisms within the PRMT network. Last but not the least, the therapeutic strategy of proteolysis-targeting chimeras (PROTACs), which entails chimeric bifunctional small molecules to recruit an E3 ligase to selectively degrade a target protein, has also been exploited on PRMT degradation (169). By linking specific PRMT inhibitors with E3 ligase ligands, PRMT-targeting PROTACs facilitate the ubiquitination and subsequent proteasomal degradation of PRMTs. This approach not only ensures sustained target depletion and the ability to target nonenzymatic functions of PRMTs but also allows for potentially lower therapeutic doses due to the catalytic turnover of PROTAC molecules. The development of PRMT-targeting PROTACs could overcome limitations of traditional inhibitors, such as compensatory upregulation and off-target effects, thereby broadening the application and enhancing the efficacy of PRMT inhibitors in treating diseases driven by dysregulated arginine methylation, especially cancers.

Currently, several MTA-dependent PRMT5 inhibitors are undergoing clinical trials for the treatment of advanced solid tumors or hematologic malignancies. These inhibitors are designed to selectively target the MTA-bound state of PRMT5, which is believed to be prevalent in MTAP-deficient tumor cells. By preferentially inhibiting PRMT5 in such a specific context, these drugs can reduce the methylation of oncogenic substrates, thereby disrupting essential cancer cell processes such as RNA splicing, transcription, and cell cycle progression. The development of MTA-dependent PRMT5 inhibitors is a rapidly advancing area of cancer therapy, with multiple promising agents showing potential in clinical settings. Notable MTA-cooperative PRMT5 inhibitors currently in clinical trials include TNG908, TNG462, AMG193, and MRTX1719, all of which have demonstrated significant antitumor efficacy in cancers harboring MTAP-deletions (118, 119, 170, 171). These inhibitors leverage the synthetic lethal relationship between PRMT5 inhibition and MTAP deficiency, allowing for a more targeted approach to cancer treatment that minimizes damage to normal, MTAP-proficient cells. As research progresses, these agents may offer a precise medicine strategy for malignancies with limited treatment options, potentially improving prognostic outcomes in patients.

Although many of the developed PRMT inhibitors show significant potency in preclinical models, more extensive clinical trials are needed to assess their efficacy and safety profiles in humans. Except for the availability of MTA-cooperative inhibitors, several other types of PRMT5 inhibitors—including JNJ-64619178, PF-06939999, PRT543, and PRT811—are currently under investigation in clinical trials for their potential efficacy against advanced solid tumors and hematologic malignancies. These inhibitors have shown preliminary efficacy, including partial or complete responses, and stable diseases in different cancers such as ACC, GBM, and uveal melanoma. Common adverse effects include thrombocytopenia, anemia, neutropenia, fatigue, and gastrointestinal symptoms (128). Notably, the phase 1 clinical trial for GSK3368715, the first PRMT1 inhibitor to enter the clinic, was terminated early due to a lack of clinical efficacy and extensive treatment-emergent effects. This underscores the multifaceted complexities in PRMT drug discovery and stress the need for extensive and cautious measures when advancing top leads to clinical tests in patients (99).

In closing, our review demonstrates that PRMT inhibitors have broad therapeutic applicability across a wide range of biomedical contexts. Some of these inhibitors have already been shown to possess great promise in the treatment of cardiovascular disorders, neurodegenerative diseases, and multiple types of cancers, including multiple myeloma, leukemia, and breast cancer, among others. By targeting dysregulated activities and/or expression of PRMTs, small molecule drugs can modulate arginine methylation-regulated cellular pathways essential to disease incidence and progression, thus offering a novel and potent strategy for managing different pathological conditions. This is an invigorating field of research and discovery. We would expect to witness more new structural chemotypes for potent and selective PRMT inhibition as well as more diverse applications of PRMT inhibitors in combating human ailments.

## Supporting information

This article contains supporting information.

## Conflict of interest

The authors declare that they have no conflicts of interest with the contents of this article.

## References

[1] Guccione E., Richard S. (2019). The regulation, functions and clinical relevance of arginine methylation. Nat. Rev. Mol. Cell Bio..

[2] Zhu Y., Yu C., Zhuang S.G. (2020). Protein arginine methyltransferase 1 mediates renal fibroblast activation and fibrogenesis through activation of Smad3 signaling. Am. J. Physiol-Renal..

[3] Strahl B.D., Allis C.D. (2000). The language of covalent histone modifications. Nature.

[4] Jenuwein T., Allis C.D. (2001). Translating the histone code. Science.

[5] Fulton M.D., Zhang J., He M., Ho M.C., Zheng Y.G. (2017). Intricate effects of alpha-amino and lysine modifications on arginine methylation of the N-terminal tail of histone H4. Biochemistry.

[6] Asatoor A.M., Armstrong M.D. (1967). 3-Methylhistidine a component of actin. Biochem. Bioph. Res. Co..

[7] Carlson S.M., Gozani O. (2016). Nonhistone lysine methylation in the regulation of cancer pathways. Cold Spring Harb. Perspect. Med..

[8] Clarke S.G. (2013). Protein methylation at the surface and buried deep: thinking outside the histone box. Trends Biochem. Sci..

[9] Johnson P., Harris C.I., Perry S.V. (1967). 3-Methylhistidine in actin and other muscle proteins. Biochem. J..

[10] Lee H.W., Kim S., Paik W.K. (1977). S-adenosylmethionine: protein-arginine methyltransferase. Purification and mechanism of the enzyme. Biochemistry.

[11] Zurita-Lopez C.I., Sandberg T., Kelly R., Clarke S.G. (2012). Human protein arginine methyltransferase 7 (PRMT7) is a type III enzyme forming ω-*NG*-monomethylated arginine residues. J. Biol. Chem..

[12] Morales Y., Caceres T., May K., Hevel J.M. (2016). Biochemistry and regulation of the protein arginine methyltransferases (PRMTs). Arch. Biochem. Biophys..

[13] Yang Y.Z., Bedford M.T. (2013). Protein arginine methyltransferases and cancer. Nat. Rev. Cancer.

[14] Bedford M.T., Richard S. (2005). Arginine methylation: an emerging regulator of protein function. Mol. Cell.

[15] Tang J., Gary J.D., Clarke S., Herschman H.R. (1998). PRMT 3, a type I protein arginine N-methyltransferase that differs from PRMT1 in its oligomerization, subcellular localization, substrate specificity, and regulation. J. Biol. Chem..

[16] Teyssier C., Chen D.G., Stallcup M.R. (2002). Requirement for multiple domains of the protein arginine methyltransferase CARM1 in its transcriptional coactivator function. J. Biol. Chem..

[17] Antonysamy S., Bonday Z., Campbell R.M., Doyle B., Druzina Z., Gheyi T. (2012). Crystal structure of the human PRMT5:MEP50 complex. P. Natl. Acad. Sci. U. S. A..

[18] Yang Y.Z., Hadjikyriacou A., Xia Z., Gayatri S., Kim D., Zurita-Lopez C. (2015). PRMT9 is a Type II methyltransferase that methylates the splicing factor SAP145. Nat. Commun..

[19] Lee J., Sayegh J., Daniel J., Clarke S., Bedford M.T. (2005). PRMT8, a new membrane-bound tissue-specific member of the protein arginine methyltransferase family. J. Biol. Chem..

[20] Hasegawa M., Toma-Fukai S., Kim J.D., Fukamizu A., Shimizu T. (2014). Protein arginine methyltransferase 7 has a novel homodimer-like structure formed by tandem repeats. FEBS Lett..

[21] Li S., Ali S., Duan X., Liu S., Du J., Liu C. (2018). JMJD1B demethylates H4R3me2s and H3K9me2 to facilitate gene expression for development of hematopoietic stem and progenitor cells. Cell Rep..

[22] Chang B.S., Chen Y., Zhao Y.M., Bruick R.K. (2007). JMJD6 is a histone arginine demethylase. Science.

[23] Li S.H., Ali S., Duan X.T., Liu S.B., Du J., Liu C.W. (2018). JMJD1B demethylates H4R3me2s and H3K9me2 to facilitate gene expression for development of hematopoietic stem and progenitor cells. Cell Rep..

[24] Wang Y., Wysocka J., Sayegh J., Lee Y.H., Perlin J.R., Leonelli L. (2004). Human PAD4 regulates histone arginine methylation levels via demethylimination. Science.

[25] Gayatri S., Bedford M.T. (2014). Readers of histone methylarginine marks. Biochim. Biophys. Acta.

[26] Wang Y., Bedford M.T. (2023). Effectors and effects of arginine methylation. Biochem. Soc. Trans..

[27] Su X., Zhu G., Ding X., Lee S.Y., Dou Y., Zhu B. (2014). Molecular basis underlying histone H3 lysine-arginine methylation pattern readout by Spin/Ssty repeats of Spindlin1. Genes Dev..

[28] Wang Y.L., Bedford M.T. (2023). Effectors and effects of arginine methylation. Biochem. Soc. T.

[29] Wang Y., Zhou J., He W., Fu R., Shi L., Dang N.K. (2024). SART3 reads methylarginine-marked glycine- and arginine-rich motifs. Cell Rep..

[30] Guccione E., Bassi C., Casadio F., Martinato F., Cesaroni M., Schuchlautz H. (2007). Methylation of histone H3R2 by PRMT6 and H3K4 by an MLL complex are mutually exclusive. Nature.

[31] Pal S., Baiocchi R.A., Byrd J.C., Grever M.R., Jacob S.T., Sif S. (2007). Low levels of miR-92b/96 induce PRMT5 translation and H3R8/H4R3 methylation in mantle cell lymphoma. EMBO J..

[32] Selvi B.R., Batta K., Kishore A.H., Mantelingu K., Varier R.A., Balasubramanyam K. (2010). Identification of a novel inhibitor of coactivator-associated arginine methyltransferase 1 (CARM1)-mediated methylation of histone H3 Arg-17. J. Biol. Chem..

[33] Bauer U.M., Daujat S., Nielsen S.J., Nightingale K., Kouzarides T. (2002). Methylation at arginine 17 of histone H3 is linked to gene activation. EMBO Rep..

[34] Strahl B.D., Briggs S.D., Brame C.J., Caldwell J.A., Koh S.S., Ma H. (2001). Methylation of histone H4 at arginine 3 occurs in vivo and is mediated by the nuclear receptor coactivator PRMT1. Curr. Biol..

[35] Zappacosta F., Wagner C.D., Della Pietra A., Gerhart S.V., Keenan K., Korenchuck S. (2021). A chemical acetylation-based mass spectrometry platform for histone methylation profiling. Mol. Cell Proteomics.

[36] Zheng K., Chen S., Ren Z., Wang Y. (2023). Protein arginine methylation in viral infection and antiviral immunity. Int. J. Biol. Sci..

[37] Deng X., Shao G., Zhang H.T., Li C., Zhang D., Cheng L. (2017). Protein arginine methyltransferase 5 functions as an epigenetic activator of the androgen receptor to promote prostate cancer cell growth. Oncogene.

[38] Greenblatt S.M., Liu F., Nimer S.D. (2016). Arginine methyltransferases in normal and malignant hematopoiesis. Exp. Hematol..

[39] Nicholson T.B., Chen T.P., Richard S. (2009). The physiological and pathophysiological role of PRMT1-mediated protein arginine methylation. Pharmacol. Res..

[40] Najbauer J., Johnson B.A., Young A.L., Aswad D.W. (1993). Peptides with sequences similar to Glycine, arginine-rich motifs in proteins interacting with rna are efficiently recognized by methyltransferase(S) modifying arginine in numerous proteins. J. Biol. Chem..

[41] Branscombe T.L., Frankel A., Lee J.H., Cook J.R., Yang Z.H., Pestka S. (2001). PRMT5 (Janus kinase-binding protein 1) catalyzes the formation of symmetric dimethylarginine residues in proteins. J. Biol. Chem..

[42] Cura V., Marechal N., Troffer-Charlier N., Strub J.M., van Haren M.J., Martin N.I. (2017). Structural studies of protein arginine methyltransferase 2 reveal its interactions with potential substrates and inhibitors. FEBS J..

[43] Cheng D., Cote J., Shaaban S., Bedford M.T. (2007). The arginine methyltransferase CARM1 regulates the coupling of transcription and mRNA processing. Mol. Cell.

[44] Feng Y., Maity R., Whitelegge J.P., Hadjikyriacou A., Li Z., Zurita-Lopez C. (2013). Mammalian protein arginine methyltransferase 7 (PRMT7) specifically targets RXR sites in lysine- and arginine-rich regions. J. Biol. Chem..

[45] Herrmann F., Fackelmayer F.O. (2009). Nucleo-cytoplasmic shuttling of protein arginine methyltransferase 1 (PRMT1) requires enzymatic activity. Genes Cells.

[46] Herrmann F., Pably P., Eckerich C., Bedford M.T., Fackelmayer F.O. (2009). Human protein arginine methyltransferases in vivo - distinct properties of eight canonical members of the PRMT family. J. Cell Sci..

[47] Hwang J.W., Cho Y., Bae G.U., Kim S.N., Kim Y.K. (2021). Protein arginine methyltransferases: promising targets for cancer therapy. Exp. Mol. Med..

[48] Gu X., He M., Lebedev T., Lin C.H., Hua Z.Y., Zheng G.R. (2022). PRMT1 is an important factor for medulloblastoma cell proliferation and survival. Biochem. Biophys. Rep..

[49] Choucair A., Pham T.H., Omarjee S., Jacquemetton J., Kassem L., Tredan O. (2019). The arginine methyltransferase PRMT1 regulates IGF-1 signaling in breast cancer. Oncogene.

[50] Zhong J., Cao R.X., Zu X.Y., Hong T., Yang J., Liu L. (2012). Identification and characterization of novel spliced variants of PRMT2 in breast carcinoma. FEBS J..

[51] El Messaoudi S., Fabbrizio E., Rodriguez C., Chuchana P., Fauquier L., Cheng D.H. (2006). Coactivator-associated arginine methyltransferase 1 (CARM1) is a positive regulator of the *Cyclin E1* gene. P. Natl. Acad. Sci. U. S. A..

[52] Wang L., Zhao Z., Meyer M.B., Saha S., Yu M., Guo A. (2014). CARM1 methylates chromatin remodeling factor BAF155 to enhance tumor progression and metastasis. Cancer Cell.

[53] Rengasamy M., Zhang F., Vashisht A., Song W.M., Aguilo F., Sun Y.D. (2017). The PRMT5/WDR77 complex regulates alternative splicing through ZNF326 in breast cancer. Nucleic Acids Res..

[54] Ryu J.W., Kim S.K., Son M.Y., Jeon S.J., Oh J.H., Lim J.H. (2017). Novel prognostic marker PRMT1 regulates cell growth via downregulation of CDKN1A in HCC. Oncotarget.

[55] Jeon J.Y., Lee J.S., Park E.R., Shen Y.N., Kim M.Y., Shin H.J. (2018). Protein arginine methyltransferase 5 is implicated in the aggressiveness of human hepatocellular carcinoma and controls the invasive activity of cancer cells. Oncol. Rep..

[56] Jiang H., Zhou Z., Jin S., Xu K., Zhang H., Xu J. (2018). PRMT9 promotes hepatocellular carcinoma invasion and metastasis via activating PI3K/Akt/GSK-3beta/Snail signaling. Cancer Sci..

[57] Avasarala S., Van Scoyk M., Rathinam M.K.K., Zerayesus S., Zhao X.M., Zhang W. (2015). PRMT1 is a novel regulator of epithelial-mesenchymal-transition in non-small cell lung cancer. J. Biol. Chem..

[58] Jing P.Y., Zhao N., Ye M.X., Zhang Y., Zhang Z.P., Sun J.Y. (2018). Protein arginine methyltransferase 5 promotes lung cancer metastasis via the epigenetic regulation of miR-99 family/FGFR3 signaling. Cancer Lett..

[59] Zhang S., Ma Y., Hu X., Zheng Y., Chen X. (2019). Targeting PRMT5/Akt signalling axis prevents human lung cancer cell growth. J. Cell Mol. Med..

[60] Avasarala S., Wu P.Y., Khan S.Q., Su Y.L., Van Scoyk M., Bao J.Q. (2020). PRMT6 promotes lung tumor progression via the alternate activation of tumor-associated macrophages. Mol. Cancer Res..

[61] Cheng D.Z., He Z.F., Zheng L.C., Xie D.Y., Dong S.W., Zhang P. (2018). Contributes to the metastasis phenotype in human non-small-cell lung cancer cells possibly through the interaction with *HSPA5* and *EEF2*. Oncotargets Ther..

[62] Liao H.W., Hsu J.M., Xia W., Wang H.L., Wang Y.N., Chang W.C. (2015). PRMT1-mediated methylation of the EGF receptor regulates signaling and cetuximab response. J. Clin. Invest..

[63] Hartley A.V., Wang B.L., Mundade R., Jiang G.L., Sun M.Y., Wei H. (2020). PRMT5-mediated methylation of YBX1 regulates NF-κB activity in colorectal cancer. Sci. Rep..

[64] Ou C.Y., LaBonte M.J., Manegold P.C., So A.Y., Ianculescu I., Gerke D.S. (2011). A coactivator role of CARM1 in the dysregulation of beta-catenin activity in colorectal cancer cell growth and gene expression. Mol. Cancer Res..

[65] Chung J., Karkhanis V., Baiocchi R.A., Sif S. (2019). Protein arginine methyltransferase 5 (PRMT5) promotes survival of lymphoma cells via activation of WNT/beta-catenin and AKT/GSK3beta proliferative signaling. J. Biol. Chem..

[66] Radzisheuskaya A., Shliaha P.V., Grinev V., Lorenzini E., Kovalchuk S., Shlyueva D. (2019). PRMT5 methylome profiling uncovers a direct link to splicing regulation in acute myeloid leukemia. Nat. Struct. Mol. Biol..

[67] Wang L., Pal S., Sif S. (2008). Protein arginine methyltransferase 5 suppresses the transcription of the RB family of tumor suppressors in leukemia and lymphoma cells. Mol. Cell Biol..

[68] Cheng D.H., Yadav N., King R.W., Swanson M.S., Weinstein E.J., Bedford M.T. (2004). Small molecule regulators of protein arginine methyltransferases. J. Biol. Chem..

[69] Fulton M.D., Brown T., Zheng Y.G. (2018). Mechanisms and inhibitors of histone arginine methylation. Chem. Rec..

[70] Hu H., Qian K., Ho M.C., Zheng Y.G. (2016). Small molecule inhibitors of protein arginine methyltransferases. Expert Opin. Investig. Drugs.

[71] Kaniskan H.U., Martini M.L., Jin J. (2018). Inhibitors of protein methyltransferases and demethylases. Chem. Rev..

[72] Wu Q., Schapira M., Arrowsmith C.H., Barsyte-Lovejoy D. (2021). Protein arginine methylation: from enigmatic functions to therapeutic targeting. Nat. Rev. Drug Discov..

[73] Yan L., Yan C., Qian K., Su H., Kofsky-Wofford S.A., Lee W.C. (2014). Diamidine compounds for selective inhibition of protein arginine methyltransferase 1. J. Med. Chem..

[74] Kaniskan H.U., Szewczyk M.M., Yu Z., Eram M.S., Yang X., Schmidt K. (2015). A potent, selective and cell-active allosteric inhibitor of protein arginine methyltransferase 3 (PRMT3). Angew. Chem. Int. Ed. Engl..

[75] Ferreira de Freitas R., Ivanochko D., Schapira M. (2019). Methyltransferase inhibitors: competing with, or exploiting the bound cofactor. Molecules.

[76] Li K.K., Luo C., Wang D., Jiang H., Zheng Y.G. (2012). Chemical and biochemical approaches in the study of histone methylation and demethylation. Med. Res. Rev..

[77] Castellano S., Spannhoff A., Milite C., Dal Piaz F., Cheng D., Tosco A. (2012). Identification of small-molecule enhancers of arginine methylation catalyzed by coactivator-associated arginine methyltransferase 1. J. Med. Chem..

[78] Feng Y., Xie N., Wu J., Yang C., Zheng Y.G. (2009). Inhibitory study of protein arginine methyltransferase 1 using a fluorescent approach. Biochem. Biophys. Res. Commun..

[79] Wu J., Xie N., Feng Y., Zheng Y.G. (2012). Scintillation proximity assay of arginine methylation. J. Biomol. Screen.

[80] Feng Y., Li M., Wang B., Zheng Y.G. (2010). Discovery and mechanistic study of a class of protein arginine methylation inhibitors. J. Med. Chem..

[81] Sun Q.Z., Yang X.D., Zhong B., Jiao F.F., Li C.Y., Li D.M. (2012). Upregulated protein arginine methyltransferase 1 by IL-4 increases eotaxin-1 expression in airway epithelial cells and participates in antigen-induced pulmonary inflammation in rats. J. Immunol..

[82] Sun Q.Z., Liu L., Roth M., Tian J., He Q.R., Zhong B. (2015). PRMT1 upregulated by epithelial proinflammatory cytokines participates in COX2 expression in fibroblasts and chronic antigen-induced pulmonary inflammation. J. Immunol..

[83] Zhang B.L., Dong S.H., Zhu R.M., Hu C.Y., Hou J., Li Y. (2015). Targeting protein arginine methyltransferase 5 inhibits colorectal cancer growth by decreasing arginine methylation of eIF4E and FGFR3. Oncotarget.

[84] Bajbouj K., Ramakrishnan R.K., Saber-Ayad M., Omar H.A., Sharif-Askari N.S., Shafarin J. (2021). PRMT5 selective inhibitor enhances therapeutic efficacy of cisplatin in lung cancer cells. Int. J. Mol. Sci..

[85] Dong S.H., Wang X., Tian S.C., Ma N.Q., Zhang X.Y., Liu Y.P. (2018). Arginine methyltransferase inhibitor 1 exhibits antitumor effects against cervical cancer in vitro and in vivo. Pharmazie.

[86] Zhang B.L., Chen X., Ge S.Y., Peng C.L., Zhang S., Chen X. (2018). Arginine methyltransferase inhibitor-1 inhibits sarcoma viability in vitro and in vivo. Oncol. Lett..

[87] Janisiak J., Kopytko P., Tkacz M., Roginska D., Peruzynska M., Machalinski B. (2021). Protein arginine methyltransferase (PRMT) inhibitors-AMI-1 and SAH are effective in attenuating rhabdomyosarcoma growth and proliferation in cell cultures. Int. J. Mol. Sci..

[88] Eram M.S., Shen Y.D., Szewczyk M.M., Wu H., Senisterra G., Li F.L. (2016). A potent, selective, and cell-active inhibitor of human type I protein arginine methyltransferases. Acs Chem. Biol..

[89] Wu Q., Nie D.Y., Ba-alawi W., Ji Y.S., Zhang Z.W., Cruickshank J. (2022). PRMT inhibition induces a viral mimicry response in triple-negative breast cancer. Nat. Chem. Biol..

[90] Hu G.H., Yan C., Xie P.Y., Cao Y., Shao J., Ge J. (2020). PRMT2 accelerates tumorigenesis of hepatocellular carcinoma by activating Bcl2 via histone H3R8 methylation. Exp. Cell Res..

[91] Plotnikov A., Kozer N., Cohen G., Carvalho S., Duberstein S., Almog O. (2020). PRMT1 inhibition induces differentiation of colon cancer cells. Sci. Rep..

[92] Dominici C., Sgarioto N., Yu Z.B., Sesma-Sanz L., Masson J.Y., Richard S. (2021). Synergistic effects of type I PRMT and PARP inhibitors against non-small cell lung cancer cells. Clin. Epigenetics.

[93] Zhang S.Y., Guo L., Zhang Z.W., Liu X.Y., Chen W.J., Wei Y. (2024). Type-I protein arginine methyltransferase inhibition primes anti-programmed cell death protein 1 immunotherapy in triple-negative breast cancer. Cancer-Am Cancer Soc..

[94] Kordala A.J., Stoodley J., Ahlskog N., Hanifi M., Garcia Guerra A., Bhomra A. (2023). PRMT inhibitor promotes SMN2 exon 7 inclusion and synergizes with nusinersen to rescue SMA mice. EMBO Mol. Med..

[95] Cai T., Yu Z.B., Wang Z., Liang C., Richard S. (2021). Arginine methylation of SARS-Cov-2 nucleocapsid protein regulates RNA binding, its ability to suppress stress granule formation, and viral replication. J. Biol. Chem..

[96] Fedoriw A., Rajapurkar S.R., O'Brien S., Gerhart S.V., Mitchell L.H., Adams N.D. (2019). Anti-tumor activity of the type I PRMT inhibitor, GSK3368715, synergizes with PRMT5 inhibition through MTAP loss. Cancer Cell.

[97] Dhar S., Vemulapalli V., Patananan A.N., Huang G.L., Di Lorenzo A., Richard S. (2013). Loss of the major Type I arginine methyltransferase PRMT1 causes substrate scavenging by other PRMTs. Sci. Rep..

[98] Liu H., Chen X., Wang P., Chen M., Deng C., Qian X. (2024). PRMT1-mediated PGK1 arginine methylation promotes colorectal cancer glycolysis and tumorigenesis. Cell Death Dis..

[99] El-Khoueiry A.B., Clarke J., Neff T., Crossman T., Ratia N., Rathi C. (2023). Phase 1 study of GSK3368715, a type I PRMT inhibitor, in patients with advanced solid tumors. Br. J Cancer.

[100] Bissinger E.M., Heinke R., Spannhoff A., Eberlin A., Metzger E., Cura V. (2011). Acyl derivatives of p-aminosulfonamides and dapsone as new inhibitors of the arginine methyltransferase hPRMT1. Bioorg. Med. Chem..

[101] Kim E., Jang J., Park J.G., Kim K.H., Yoon K., Yoo B.C. (2020). Protein arginine methyltransferase 1 (PRMT1) selective inhibitor, TC-E 5003, has anti-inflammatory properties in TLR4 signaling. Int. J. Mol. Sci..

[102] Zhang P., Tao H., Yu L., Zhou L., Zhu C. (2020). Developing protein arginine methyltransferase 1 (PRMT1) inhibitor TC-E-5003 as an antitumor drug using INEI drug delivery systems. Drug Deliv..

[103] Park M.J., Liao J., Kim D.I. (2020). TC-E 5003, a protein methyltransferase 1 inhibitor, activates the PKA-dependent thermogenic pathway in primary murine and human subcutaneous adipocytes. FEBS Lett..

[104] Qian K., Yan C., Su H., Dang T., Zhou B., Wang Z. (2021). Pharmacophore-based screening of diamidine small molecule inhibitors for protein arginine methyltransferases. RSC Med. Chem..

[105] Zhang J., Qian K., Yan C.L., He M.M., Jassim B.A., Ivanov I. (2017). Discovery of decamidine as a new and potent PRMT1 inhibitor. Med. Chem. Comm..

[106] Kim S.W., Ahn B.Y., Tran T.T.V., Pyun J.H., Kang J.S., Leem Y.E. (2022). PRMT1 suppresses doxorubicin-induced cardiotoxicity by inhibiting endoplasmic reticulum stress. Cell Signal.

[107] Yan C.L., Yan L.L., Qian K., Zhao X.Y., Zheng Y.G., Ivanov I. (2014). Diamidine compounds for selective inhibition of protein arginine methyltransferase 1. Abstr. Pap. Am. Chem. S.

[108] Xu J., Wang A.H.J., Oses-Prieto J., Makhijani K., Katsuno Y., Pei M. (2013). Arginine methylation initiates BMP-induced Smad signaling. Mol. Cell.

[109] Zhang L., Tran N.T., Su H., Wang R., Lu Y., Tang H. (2015). Cross-talk between PRMT1-mediated methylation and ubiquitylation on RBM15 controls RNA splicing. Elife.

[110] Siboni R.B., Bodner M.J., Khalifa M.M., Docter A.G., Choi J.Y., Nakamori M. (2015). Biological efficacy and toxicity of diamidines in myotonic dystrophy type 1 models. J. Med. Chem..

[111] Samuel S.F., Marsden A.J., Deepak S., Rivero F., Greenman J., Beltran-Alvarez P. (2018). Inhibiting arginine methylation as a tool to investigate cross-talk with methylation and acetylation post-translational modifications in a glioblastoma cell line. Proteomes.

[112] Jiang L.C., Liao J., Liu J.H., Wei Q.Z., Wang Y.Y. (2021). Geranylgeranylacetone promotes human osteosarcoma cell apoptosis by inducing the degradation of PRMT1 through the E3 ubiquitin ligase CHIP. J. Cell Mol. Med..

[113] Hua Z.Y., Hansen J.N., He M., Dai S.K., Choi Y., Fulton M.D. (2020). PRMT1 promotes neuroblastoma cell survival through ATF5. Oncogenesis.

[114] Lee Y.J., Chang W.W., Chang C.P., Liu T.Y., Chuang C.Y., Qian K. (2019). Downregulation of PRMT1 promotes the senescence and migration of a non- amplified neuroblastoma SK-N-SH cells. Sci. Rep..

[115] Mavrakis K.J., McDonald E. R., 3rd, Schlabach M.R., Billy E., Hoffman G.R., deWeck A. (2016). Disordered methionine metabolism in MTAP/CDKN2A-deleted cancers leads to dependence on PRMT5. Science.

[116] Kryukov G.V., Wilson F.H., Ruth J.R., Paulk J., Tsherniak A., Marlow S.E. (2016). MTAP deletion confers enhanced dependency on the PRMT5 arginine methyltransferase in cancer cells. Science.

[117] Marjon K., Cameron M.J., Quang P., Clasquin M.F., Mandley E., Kunii K. (2016). MTAP deletions in cancer create vulnerability to targeting of the MAT2A/PRMT5/RIOK1 Axis. Cell Rep..

[118] Lynch J.T., Moore S., Barrantes I.D., Bradshaw L., Chambers C., Hong T. (2023). AZ-PRMT5i-1: a potent MTAP-selective PRMT5 inhibitor with pharmacodynamic and monotherapy anti-tumor activity in MTAP-deleted tumours. Cancer Res..

[119] Engstrom L.D., Aranda R., Waters L., Moya K., Bowcut V., Vegar L. (2023). MRTX1719 is an MTA-cooperative PRMT5 inhibitor that exhibits synthetic lethality in preclinical models and patients with MTAP-deleted cancer. Cancer Discov..

[120] Chan-Penebre E., Kuplast K.G., Majer C.R., Boriack-Sjodin P.A., Wigle T.J., Johnston L.D. (2015). A selective inhibitor of PRMT5 with in vivo and in vitro potency in MCL models. Nat. Chem. Biol..

[121] Liu X., He J.Z., Mao L.B., Zhang Y.Y., Cui W.W., Duan S.J. (2021). EPZ015666, a selective protein arginine methyltransferase 5 (PRMT5) inhibitor with an antitumour effect in retinoblastoma. Exp. Eye Res..

[122] Jiang Y., Zheng G., Sun X. (2023). PRMT5 promotes retinoblastoma development. Hum. Cell.

[123] Chaturvedi N.K., Mahapatra S., Kesherwani V., Kling M.J., Shukla M., Ray S. (2019). Role of protein arginine methyltransferase 5 in group 3 (MYC-driven) Medulloblastoma. BMC Cancer.

[124] Braun C.J., Stanciu M., Boutz P.L., Patterson J.C., Calligaris D., Higuchi F. (2017). Coordinated splicing of regulatory detained introns within oncogenic transcripts creates an exploitable vulnerability in malignant glioma. Cancer Cell.

[125] Gao G.Z., Dhar S., Bedford M.T. (2017). PRMT5 regulates IRES-dependent translation via methylation of hnRNP A1. Nucleic Acids Res..

[126] Araujo-Abad S., Manresa-Manresa A., Rodriguez-Canas E., Fuentes-Baile M., Garcia-Morales P., Mallavia R. (2023). Glioblastoma-derived small extracellular vesicles: nanoparticles for glioma treatment. Int. J. Mol. Sci..

[127] Vinet M., Suresh S., Maire V., Monchecourt C., Nemati F., Lesage L. (2019). Protein arginine methyltransferase 5: a novel therapeutic target for triple-negative breast cancers. Cancer Med..

[128] Feustel K., Falchook G.S. (2022). Protein arginine methyltransferase 5 (PRMT5) inhibitors in oncology clinical trials: a review. J. Immunother. Precis Oncol..

[129] Shifteh D., Sapir T., Goel S., Maitra R. (2020). Protein arginine methyltransferase 5 as a therapeutic target for KRAS mutated colorectal cancer. Cancer Res..

[130] Gulla A., Hideshima T., Bianchi G., Fulciniti M., Samur M.K., Qi J. (2018). Protein arginine methyltransferase 5 has prognostic relevance and is a druggable target in multiple myeloma. Leukemia.

[131] Duncan K.W., Rioux N., Boriack-Sjodin P.A., Munchhof M.J., Reiter L.A., Majer C.R. (2016). Structure and property guided design in the identification of PRMT5 tool compound EPZ015666. ACS Med. Chem. Lett..

[132] Xia T., Liu M., Zhao Q., Ouyang J., Xu P., Chen B. (2021). PRMT5 regulates cell pyroptosis by silencing CASP1 in multiple myeloma. Cell Death Dis..

[133] Fong J.Y., Pignata L., Goy P.A., Kawabata K.C., Lee S.C.W., Koh C.M. (2019). Therapeutic targeting of RNA splicing catalysis through inhibition of protein arginine methylation. Cancer Cell.

[134] Ding M., Cho E., Chen Z., Park S.W., Lee T.H. (2023). (S)-2-(Cyclobutylamino)-N-(3-(3,4-dihydroisoquinolin-2(1H)-yl)-2-hydroxypropyl)isonicotinamide attenuates RANKL-induced osteoclast differentiation by inhibiting NF-kappaB nuclear translocation. Int. J. Mol. Sci..

[135] Sachamitr P., Ho J.C., Ciamponi F.E., Ba-Alawi W., Coutinho F.J., Guilhamon P. (2021). PRMT5 inhibition disrupts splicing and stemness in glioblastoma. Nat. Commun..

[136] Li Y., Yang Y., Liu X., Long Y., Zheng Y. (2019). PRMT5 promotes human lung cancer cell apoptosis via Akt/Gsk3beta signaling induced by resveratrol. Cell Transpl..

[137] Huang J.H., Zheng Y.H., Zheng X., Qian B., Yin Q., Lu J.J. (2021). PRMT5 promotes EMT through regulating Akt activity in human lung cancer. Cell Transplant..

[138] Bonday Z.Q., Cortez G.S., Grogan M.J., Antonysamy S., Weichert K., Bocchinfuso W.P. (2018). LLY-283, a potent and selective inhibitor of arginine methyltransferase 5, PRMT5, with antitumor activity. ACS Med. Chem. Lett..

[139] Chen Q., Xie F., Ji Y.Y., Wei K.J., Ren Q. (2022). [LLY-283 inhibits proliferation and metastasis of head and neck squamous cell carcinoma by targeting PRMT5]. Shanghai Kou Qiang Yi Xue.

[140] Kumar H., Dhir A., Paterson A.J., Anderson N.R., Qiu S.W., Zhao X.Y. (2022). PRMT5 inhibition enhances elimination of FLT3-ITD AML stem cells in combination with TKI treatment. Blood.

[141] Zhao B., Zhang D.D., Sun Y.X., Lei M., Zeng P.J., Wang Y. (2022). Explore the effect of LLY-283 on the ototoxicity of auditory cells caused by cisplatin: a bioinformatic analysis based on RNA-seq. J. Clin. Lab Anal..

[142] Liu C., Tang D.M., Zheng Z.W., Lu X.L., Li W., Zhao L.P. (2022). A PRMT5 inhibitor protects against noise-induced hearing loss by alleviating ROS accumulation. Ecotox Environ. Safe.

[143] Wu X., Wang B., Li J.X., Yang Z.B., Zhou Y.F., Ma X.D. (2022). Inhibition of PRMT5 attenuates cerebral ischemia/reperfusion-Induced inflammation and pyroptosis through suppression of NF-kappa B/NLRP3 axis. Neurosci. Lett..

[144] de Jong L.M., Zhang Z., den Hartog Y., Sijsenaar T.J.P., Martins Cardoso R., Manson M.L. (2022). PRMT3 inhibitor SGC707 reduces triglyceride levels and induces pruritus in Western-type diet-fed LDL receptor knockout mice. Sci. Rep..

[145] Lei Y., Han P., Chen Y., Wang H., Wang S., Wang M. (2022). Protein arginine methyltransferase 3 promotes glycolysis and hepatocellular carcinoma growth by enhancing arginine methylation of lactate dehydrogenase A. Clin. Transl. Med..

[146] Zhang X., Wang K., Feng X., Wang J., Chu Y., Jia C. (2021). PRMT3 promotes tumorigenesis by methylating and stabilizing HIF1alpha in colorectal cancer. Cell Death Dis..

[147] Min Z., Xiaomeng L., Zheng L., Yangge D., Xuejiao L., Longwei L. (2019). Asymmetrical methyltransferase PRMT3 regulates human mesenchymal stem cell osteogenesis via miR-3648. Cell Death Dis..

[148] Drew A.E., Moradei O., Jacques S.L., Rioux N., Boriack-Sjodin A.P., Allain C. (2017). Identification of a CARM1 inhibitor with potent in vitro and in vivo activity in preclinical models of multiple myeloma. Sci. Rep..

[149] Greenblatt S.M., Man N., Hamard P.J., Asai T., Karl D., Martinez C. (2019). CARM1 is essential for myeloid leukemogenesis but dispensable for normal hematopoiesis (vol 33, pg 1111, 2018). Cancer Cell.

[150] Lee J., Pang K., Kim J., Hong E., Lee J., Cho H.J. (2022). ESRP1-regulated isoform switching of LRRFIP2 determines metastasis of gastric cancer. Nat. Commun..

[151] Santos M., Hwang J.W., Bedford M.T. (2023). CARM1 arginine methyltransferase as a therapeutic target for cancer. J. Biol. Chem..

[152] Nakayama K., Szewczyk M.M., Dela Sena C., Wu H., Dong A., Zeng H. (2018). TP-064, a potent and selective small molecule inhibitor of PRMT4 for multiple myeloma. Oncotarget.

[153] Zhang Y.H., Verwilligen R.A.F., de Boer M., Sijsenaar T.J.P., Van Eck M., Hoekstra M. (2021). PRMT4 inhibitor TP-064 impacts both inflammatory and metabolic processes without changing the susceptibility for early atherosclerotic lesions in male apolipoprotein E knockout mice. Atherosclerosis.

[154] Shen Y., Szewczyk M.M., Eram M.S., Smil D., Kaniskan H.U., de Freitas R.F. (2016). Discovery of a potent, selective, and cell-active dual inhibitor of protein arginine methyltransferase 4 and protein arginine methyltransferase 6. J. Med. Chem..

[155] Stossi F., Dandekar R.D., Mancini M.G., Gu G.W., Fuqua S.A.W., Nardone A. (2020). Estrogen-induced transcription at individual alleles is independent of receptor level and active conformation but can be modulated by coactivators activity. Nucleic Acids Res..

[156] Liu J., Feng J., Li L., Lin L., Ji J., Lin C. (2020). Arginine methylation-dependent LSD1 stability promotes invasion and metastasis of breast cancer. EMBO Rep..

[157] Shen Y., Li F., Szewczyk M.M., Halabelian L., Park K.S., Chau I. (2020). Discovery of a first-in-class protein arginine methyltransferase 6 (PRMT6) covalent inhibitor. J. Med. Chem..

[158] Shen Y., Li F., Szewczyk M.M., Halabelian L., Chau I., Eram M.S. (2021). A first-in-class, highly selective and cell-active allosteric inhibitor of protein arginine methyltransferase 6. J. Med. Chem..

[159] Levine M.N., Raines R.T. (2012). Trimethyl lock: a trigger for molecular release in chemistry, biology, and pharmacology. Chem. Sci..

[160] Szewczyk M.M., Ishikawa Y., Organ S., Sakai N., Li F., Halabelian L. (2020). Author Correction: pharmacological inhibition of PRMT7 links arginine monomethylation to the cellular stress response. Nat. Commun..

[161] Wang B.Y., Zhang M.R., Guo J.K., Liu Z.G., Zhou R., Guo F. (2021). The effects of flavonoid apigenin on male reproductive Health: inhibition of spermatogonial proliferation through downregulation of Prmt7/Akt3 pathway. Int. J. Mol. Sci..

[162] Dai W., Zhang J., Li S., He F., Liu Q., Gong J. (2022). Protein arginine methylation: an emerging modification in cancer immunity and immunotherapy. Front. Immunol..

[163] Zhu Y., Xia T., Chen D.Q., Xiong X., Shi L., Zuo Y. (2024). Promising role of protein arginine methyltransferases in overcoming anti-cancer drug resistance. Drug Resist. Updat.

[164] Yang Y., Bedford M.T. (2013). Protein arginine methyltransferases and cancer. Nat. Rev. Cancer.

[165] Xu J., Richard S. (2021). Cellular pathways influenced by protein arginine methylation: implications for cancer. Mol. Cell.

[166] Pak M.L., Lakowski T.M., Thomas D., Vhuiyan M.I., Husecken K., Frankel A. (2011). A protein arginine N-methyltransferase 1 (PRMT1) and 2 heteromeric interaction increases PRMT1 enzymatic activity. Biochemistry.

[167] Cao M.T., Feng Y., Zheng Y.G. (2023). Protein arginine methyltransferase 6 is a novel substrate of protein arginine methyltransferase 1. World J. Biol. Chem..

[168] Nie M., Wang Y., Guo C., Li X., Wang Y., Deng Y. (2018). CARM1-mediated methylation of protein arginine methyltransferase 5 represses human gamma-globin gene expression in erythroleukemia cells. J. Biol. Chem..

[169] Martin P.L., Pérez-Areales F.J., Rao S.V., Walsh S.J., Carroll J.S., Spring D.R. (2024). Towards the targeted protein degradation of PRMT1. Chem. Med. Chem..

[170] Cottrell K.M., Briggs K.J., Whittington D.A., Jahic H., Ali J.A., Davis C.B. (2024). Discovery of TNG908: a selective, brain penetrant, MTA-cooperative PRMT5 inhibitor that is synthetically lethal with MTAP-deleted cancers. J. Med. Chem..

[171] Calero M.V., Patnaik A., Maki R., O'Neil B., Abbruzzese J., Dagogo-Jack I. (2022). Design of a phase 1 study of AMG 193, an MTA-cooperative PRMT5 inhibitor, in patients with advanced MTAP-null solid tumors. J. Thorac. Oncol..

