# Supplementary material for: Biomedical effects of protein arginine methyltransferase inhibitors
Source: J Biol Chem. 2025 Jan 16;301(3):108201. doi: 10.1016/j.jbc.2025.108201 (PMC11871472; doi:10.1016/j.jbc.2025.108201)
Supplement: Supplementary file [file mmc1.pdf]

Supplementary Table S1. Histone substrate specificity of PRMTs.

PRMT members	Histone methylations sites	References
PRMT1	H2AR3me2a<br>H2AR11me2a<br>H4R3me2a	(1)<br>(2)<br>(3)
PRMT2	H3R8me2a	(4)
PRMT3	H4R3me2a	(5)
CARM1	H3R2me2a<br>H3R17me2a<br>H3R26me2a<br>H3R42me2a	(6)<br>(7)<br>(7)<br>(8)
PRMT5	H2AR3me1/ H2AR3me2s<br>H3R2me1/H3R2me2s<br>H3R8me2s<br>H4R3me2s	(9)<br>(10)<br>(11)<br>(11)
PRMT6	H2AR3me2a<br>H2AR11me2a<br>H2AR29me2a<br>H3R2me2a<br>H3R42me2a<br>H4R3me2a	(12)<br>(2)<br>(2)<br>(12,13)<br>(8)<br>(13)
PRMT7	H2AR3me1<br>H2BR29me1<br>H2BR31me1<br>H2BR33me1<br>H4R3me1<br>H4R17me1<br>H4R19me1	(14)<br>(7)<br>(7)<br>(7)<br>(15)<br>(16)<br>(16)
PRMT8	H4R3me2a	(17)

References

1. Strahl, B. D., Briggs, S. D., Brame, C. J., Caldwell, J. A., Koh, S. S., Ma, H., Cook, R. G., Shabanowitz, J., Hunt, D. F., Stallcup, M. R., and Allis, C. D. (2001) Methylation of histone H4 at arginine 3 occurs in vivo and is mediated by the nuclear receptor coactivator PRMT1. *Current Biology* **11**, 996-1000
2. Waldmann, T., Izzo, A., Kamieniarz, K., Richter, F., Vogler, C., Sarg, B., Lindner, H., Young, N. L., Mittler, G., Garcia, B. A., and Schneider, R. (2011) Methylation of H2AR29 is a novel repressive PRMT6 target. *Epigenetics & Chromatin* **4**
3. Wang, H. B., Huang, Z. Q., Xia, L., Feng, Q., Erdjument-Bromage, H., Strahl, B. D., Briggs, S. D., Allis, C. D., Wong, J. M., Tempst, P., and Zhang, Y. (2001) Methylation

of histone H4 at arginine 3 facilitating transcriptional activation by nuclear hormone receptor. *Science* **293**, 853-857
4. Dong, F., Li, Q., Yang, C., Huo, D. W., Wang, X., Ai, C. B., Kong, Y., Sun, X. Y., Wang, W., Zhou, Y., Liu, X., Li, W., Gao, W. W., Liu, W., Kang, C. S., and Wu, X. D. (2018) PRMT2 links histone H3R8 asymmetric dimethylation to oncogenic activation and tumorigenesis of glioblastoma. *Nature Communications* **9**
5. Zhang, M., Liu, X. M., Li, Z., Du, Y. G., Liu, X. J., Lv, L. W., Zhang, X., Liu, Y. S., Zhang, P., and Zhou, Y. S. (2019) Asymmetrical methyltransferase PRMT3 regulates human mesenchymal stem cell osteogenesis via miR-3648. *Cell Death & Disease* **10**
6. Torres-Padilla, M. E., Parfitt, D. E., Kouzarides, T., and Zernicka-Goetz, M. (2007) Histone arginine methylation regulates pluripotency in the early mouse embryo. *Nature* **445**, 214-218
7. Feng, Y., Maity, R., Whitelegge, J. P., Hadjikyriacou, A., Li, Z. W., Zurita-Lopez, C., Al-Hadid, Q., Clark, A. T., Bedford, M. T., Masson, J. Y., and Clarke, S. G. (2013) Mammalian Protein Arginine Methyltransferase 7 (PRMT7) Specifically Targets R Sites in Lysine- and Arginine-rich Regions. *Journal of Biological Chemistry* **288**, 37010-37025
8. Casadio, F., Lu, X. D., Pollock, S. B., LeRoy, G., Garcia, B. A., Muir, T. W., Roeder, R. G., and Allis, C. D. (2013) H3R42me2a is a histone modification with positive transcriptional effects. *Proc Natl Acad Sci U S A* **110**, 14894-14899
9. Pollack, B. P., Kotenko, S. V., He, W., Izotova, L. S., Barnoski, B. L., and Pestka, S. (1999) The human homologue of the yeast proteins Skb1 and Hsl7p interacts with Jak kinases and contains protein methyltransferase activity. *Journal of Biological Chemistry* **274**, 31531-31542
10. Migliori, V., Muller, J., Phalke, S., Low, D., Bezzi, M., Mok, W. C., Sahu, S. K., Gunaratne, J., Capasso, P., Bassi, C., Cecatiello, V., De Marco, A., Blackstock, W., Kuznetsov, V., Amati, B., Mapelli, M., and Guccione, E. (2012) Symmetric dimethylation of H3R2 is a newly identified histone mark that supports euchromatin maintenance. *Nat Struct Mol Biol* **19**, 136-144
11. Pal, S., Vishwanath, S. N., Erdjument-Bromage, H., Tempst, P., and Sif, S. (2004) Human SWI/SNF-associated PRMT5 methylates histone H3 arginine 8 and negatively regulates expression of and tumor suppressor genes. *Molecular and Cellular Biology* **24**, 9630-9645
12. Hyllus, D., Stein, C., Schnabel, K., Schiltz, E., Imhof, A., Dou, Y., Hsieh, J., and Bauer, U. M. (2007) PRMT6-mediated methylation of R2 in histone H3 antagonizes H3 K4 trimethylation. *Genes Dev* **21**, 3369-3380
13. Guccione, E., Bassi, C., Casadio, F., Martinato, F., Cesaroni, M., Schuchlautz, H., Lüscher, B., and Amati, B. (2007) Methylation of histone H3R2 by PRMT6 and H3K4 by an MLL complex are mutually exclusive. *Nature* **449**, 933-U918
14. Karkhanis, V., Wang, L., Tae, S., Hu, Y. J., Imbalzano, A. N., and Sif, S. (2012) Protein arginine methyltransferase 7 regulates cellular response to DNA damage by methylating promoter histones H2A and H4 of the polymerase delta catalytic subunit gene, POLD1. *J Biol Chem* **287**, 29801-29814

15. Karkhanis, V., Wang, L., Tae, S., Hu, Y. J., Imbalzano, A. N., and Sif, S. (2012) Protein Arginine Methyltransferase 7 Regulates Cellular Response to DNA Damage by Methylating Promoter Histones H2A and H4 of the Polymerase δ Catalytic Subunit Gene,. *Journal of Biological Chemistry* **287**, 29801-29814
16. Halabelian, L., and Barsyte-Lovejoy, D. (2021) Structure and Function of Protein Arginine Methyltransferase PRMT7. *Life-Basel* **11**
17. Lee, J., Sayegh, J., Daniel, J., Clarke, S., and Bedford, M. T. (2005) PRMT8, a new membrane-bound tissue-specific member of the protein arginine methyltransferase family. *J Biol Chem* **280**, 32890-32896
